# A Whole-Body Musculoskeletal Model of the Mouse

**DOI:** 10.1109/access.2021.3133078

**Published:** 2021-12-06

**Authors:** Shravan Tata Ramalingasetty, Simon M. Danner, Jonathan Arreguit, Sergey N. Markin, Dimitri Rodarie, Claudia Kathe, Grégoire Courtine, Ilya A. Rybak, Auke Jan Ijspeert

**Affiliations:** 1 Biorobotic Laboratory (BioRob), School of Engineering, Institute of Bioengineering, École Polytechnique Fédérale de Lausanne, 1015 Lausanne, Switzerland; 2 Department of Neurobiology and Anatomy, College of Medicine, Drexel University, Philadelphia, PA 19104, USA; 3 BBP-CORE, Campus Biotech, École Polytechnique Fédérale de Lausanne, 1202 Geneva, Switzerland; 4 Center for Neuroprosthetics and Brain Mind Institute, School of Life Sciences, École Polytechnique Fédérale de Lausanne, 1015 Lausanne, Switzerland

**Keywords:** Mouse, musculoskeletal, biomechanical, moment-arms, open-source model, biomechanics, neuromechanical

## Abstract

Neural control of movement cannot be fully understood without careful consideration of interactions between the neural and biomechanical components. Recent advancements in mouse molecular genetics allow for the identification and manipulation of constituent elements underlying the neural control of movement. To complement experimental studies and investigate the mechanisms by which the neural circuitry interacts with the body and the environment, computational studies modeling motor behaviors in mice need to incorporate a model of the mouse musculoskeletal system. Here, we present the first fully articulated musculoskeletal model of the mouse. The mouse skeletal system has been developed from anatomical references and includes the sets of bones in all body compartments, including four limbs, spine, head and tail. Joints between all bones allow for simulation of full 3D mouse kinematics and kinetics. Hindlimb and forelimb musculature has been implemented using Hill-type muscle models. We analyzed the mouse whole-body model and described the moment-arms for different hindlimb and forelimb muscles, the moments applied by these muscles on the joints, and their involvement in limb movements at different limb/body configurations. The model represents a necessary step for the subsequent development of a comprehensive neuro-biomechanical model of freely behaving mice; this will close the loop between the neural control and the physical interactions between the body and the environment.

## I. INTRODUCTION

**FIGURE 1.**
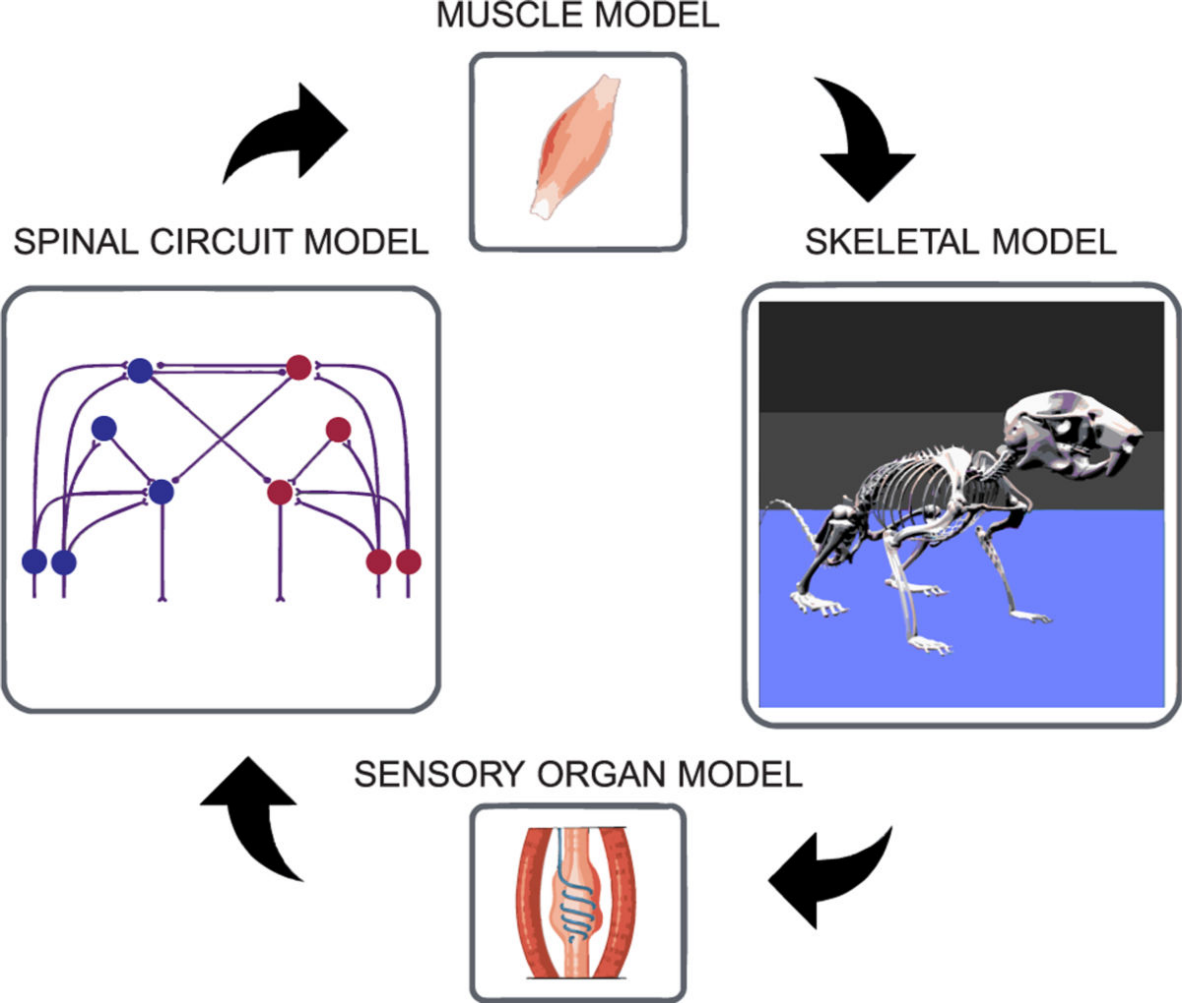
Components of a closed-loop neuromechanical simulation. Movements in animals arise due to complex interactions between the nervous system, the musculoskeletal system and the environment. A neuromechanical model includes the neural and biomechanical components along with their interactions. Neural models (spinal and brain circuits) produce the necessary instruction signals (motoneuron activations) for a specific movement. Muscle models respond to the neural signals by producing forces that act on the skeletal model and cause movements. The skeletal model and the body interact with the environment to produce reaction forces. The sensory organ models encode the state of the movement (somatosensory afferent feedback signals) and transmit this information back to the nervous system which then adapts the instruction signals to sensed perturbations or external forces.

Terrestrial animals exhibit a variety of complex motor behaviors, some rhythmic (e.g., locomotion, grooming) and some non-rhythmic (e.g., reaching and grasping, crouching, posture-control). These motor behaviors result from complex interactions between the neural circuits in the brain and spinal cord, the musculoskeletal system, and the environment (Fig. 1) [1]–[5]. Investigating the underlying mechanisms of these motor behaviors in awake, behaving animals is highly challenging. Computational modeling is a powerful tool to complement experimental studies of neural control. It provides a platform to investigate the underlying neural mechanisms, allowing investigators to reproduce existing experimental observations, propose mechanistic explanations for the observed behaviors, suggest new experiments to test the proposed mechanisms, and propose possible approaches for treatment of different injuries or disorders [6], [7].

Recent advances in mouse molecular genetic approaches have led to substantial progress in the studies of spinal and supra-spinal networks involved in motor control [2], [7]–[15]. Specifically, it is now possible to dissect the neural network by identifying and manipulating specific neural populations and to relate them to specific behavioral outcomes [2]. The ubiquity of molecular genetic tools available for the mouse made it the preferred experimental animal for the study of neural control of movement.

Computational modeling of motor control in freely behaving mice, particularly modeling of locomotion, requires the development of detailed models of both the neural and biomechanical components and, importantly, their interactions during movements [16] (Fig. 1). Such detailed musculoskeletal models have been developed of the human [17]–[21], the guinea fowl [22], the ostrich [23], and the dog [24]. Yet, no detailed whole-body three-dimensional musculoskeletal model of mouse currently exists; and most of the existing neuromechanical models (models incorporating neural controllers with the musculoskeletal system) of mammals have been limited to two-dimensional movements or had significantly simplified musculoskeletal or muscular components of the system [7], [25]–[30].

Here, we present an open-source, configurable whole-body musculoskeletal model of the mouse. We digitized all the bones of the mouse skeletal system and identified the corresponding joints to have a fully articulated rigid body model. Musculature for hindlimbs and forelimbs were modeled as Hill-type muscles. We compared and validated the hindlimb musculature of the model with the previously published single hindlimb model of mouse developed by Charles *et al.* [31]. The three-dimensional (3D) model allowed us to explore the relationship of muscle moment-arms and moments on the hindlimb and forelimb joints in a comprehensive manner. Our analysis on the operational range of muscle-fibers for limb muscles give insights into the role of active and passive forces under isometric conditions. Finally, we evaluated the sensitivities of parameters to highlight a reduced set of crucial parameters in the complex whole-body mouse model.

## II. METHODS

### A. SIMULATION TOOLS

The mouse body was simulated as articulated rigid segments interacting with the environment. We used the Bullet v3.1.7 physics engine [32]. Bullet was chosen because it is a fast and stable simulator of complex articulated rigid bodies. It employs Featherstone’s algorithm [33], which uses a reduced coordinate representation and an articulated-body inertia description to provide stability for models with long kinematic chains and links with larger mass differences. Furthermore, Bullet is available on most common operating systems and it has an application programming interface (API) for Python that allows for a full control of simulated behavior. To simulate muscle dynamics and their interactions with the skeletal system, we developed a simulator-agnostic muscle library and integrated it with Bullet.

### B. SKELETAL SYSTEM

The mouse skeletal system was represented by a set of articulated rigid segments. The construction of this model involved obtaining skeletal scans, identifying joints, and computing inertial parameters.

#### 1) DIGITIZATION

The skeletal model was based on several mouse skeletal scans and anatomical reference data. The model simulates a generic mouse based on references from several specimens. All the bones of the mouse were digitized as individual mesh objects. This included 23 tail bones, 8 cervical bones, 13 thoracic bones, 6 lumbar bones, 4 sacral (merged together as Pelvis bone), 3 coccygeal, and 20 caudal vertebrae, 15 bones in each forelimb (including digits), 13 bones in each hindlimb (including digits), the pelvis, and the head. In total, the model consists of 108 bones (Table 1). The hind and forelimb bones are modeled to be symmetric across the sagittal plane.

#### 2) IDENTIFICATION OF CENTERS FOR JOINT ROTATION

Joint rotation centers for the model were chosen such that the rotations introduced minimal interference with neighboring bones within the range-of-motion of the joint. The rotation centers remain fixed throughout the range-of-motion.

Among the tail and spinal bones, we modeled two rotational degrees-of-freedom (DoFs) between each pair of consecutive bones: flexion-extension (rotation about the transversal axis) and lateral bending (rotation about the sagittal axis).

In the hindlimbs, the hip joints were modeled as spherical joints with three rotational DoFs: flexion-extension (rotation about the transversal axis), abduction-adduction (rotation about the coronal axis), and internal-external rotation (rotation about the sagittal axis). Knee joints were modeled with a single DoF, a flexion-extension (rotation about the transversal axis) joint. Ankle joints were modeled with three DoFs: plantarflexion-dorsiflexion (rotation about the transversal axis), abduction-adduction (rotation about the coronal axis) and inversion-eversion (rotation about the sagittal axis). Only a single DoF of flexion-extension (rotation about the transversal axis) was modeled between each digit in the hindpaws.

In the forelimbs, the shoulder joints were modeled as a spherical joint with three rotational DoFs: retraction-protraction (rotation about the transversal axis), abduction-adduction (rotation about the coronal axis), and external-internal rotation (rotation about the sagittal axis). Elbow joints were modeled with two DoFs: extension-flexion (rotation about the transversal axis) and supination-pronation (rotation about the sagittal axis). Wrist joints were modeled with two DoFs: extension-flexion (rotation about the transversal axis), and abduction-adduction (rotation about the coronal axis). Only a single DoF of flexion-extension (rotation about the transversal axis) was modeled between each digit in the forepaws.

In total, the mouse model consists of 225 rotational joints. Table 1 lists the joints in the model.

#### 3) JOINT RANGE-OF-MOTION AND LIMITS

Joint range-of-motion defines the extent to which a joint can rotate between a minimal and a maximal angle. It is an important attribute for articulated rigid body simulations. For animals, joint ranges are imposed due to a combination of factors such as ligaments, muscles, and elastic forces of tissues. From previous experimental measurements on mice and rats, we have compiled and reported the joint limits for the DoFs of the model in Table 1. In this work, we modeled joint limits to mimic elastic ligaments that engage beyond the specified joint range by applying a resisting torque (modeled as a torsional spring and damper system as described in Opensim *v4.2* [34]).

#### 4) ZERO-POSE

**FIGURE 2.**
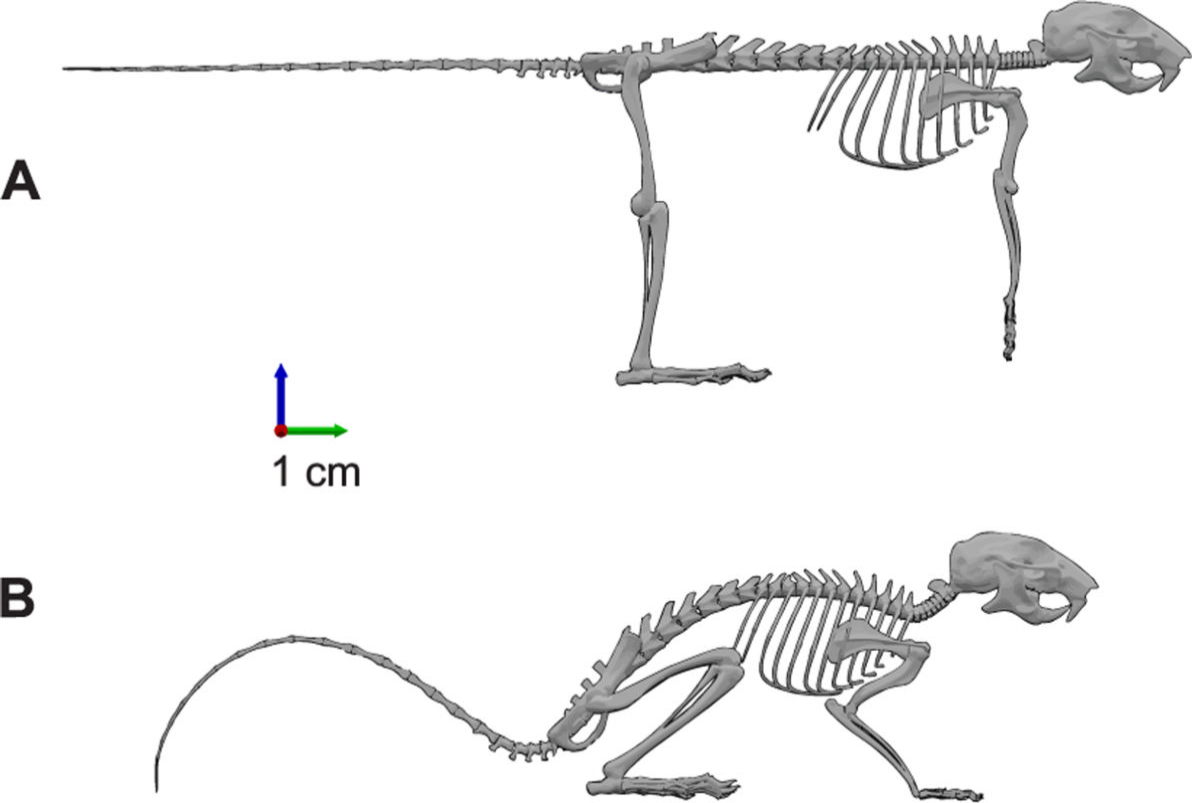
Representation of skeletal model poses. (A) The skeletal model of the mouse in zero-pose, i.e., when all the joint angles are set to zero. The zero-pose need not necessarily fall into the range-of-motion for a given joint. For example, the knee joint is defined to operate between −145.0 and −45.0 with respect to the zero position. (B) An example of the mouse in a sitting posture that is defined relative to the zero-pose.

Zero-pose defines the reference coordinate system. In the model, the zero-pose was defined as the pose at which all the joint angles are set to zero with respect to the corresponding coordinate frame. A non-anatomical pose in which some joints go beyond natural joint limits is chosen as the zero-pose to facilitate the coordinate frame transformations. Joint angles are measured with respect to the zero-pose. The model in the zero-pose is shown in Fig. 2A and a possible rest-pose is shown in Fig. 2B.

#### 5) ESTIMATING INERTIAL PROPERTIES

Inertial parameters are among the most important parameters for an accurate articulated rigid-body physics simulation. These parameters include the mass, the center-of-mass, and the inertia tensor for every bone (and surrounding soft tissues). To compute the inertial parameters, we assumed an uniform density of water (1000 kg/m^3^) along the body [39]–[43]. The volume around the bones was estimated based on the convex hulls that represent the net volume encapsulated by the skin, soft-tissues, muscles, and the bone [44], [45]. Masses were computed based on the density and volumes of the convex hulls. The center-of-mass and inertia tensors were computed based on the shape of the convex hulls and estimated mass assuming uniform density. The mouse model is 16.90 cm long from the head to the tip of the tail in the zero-pose and weighs 34.32 grams.

### C. MUSCLE SYSTEM

Muscles were modeled using the Hill-type formalism [46]. Modeling each muscle required identification of attachment points in the hind and forelimbs and the estimation of their parameters.

#### 1) HILL-TYPE MUSCLE MODEL

**FIGURE 3.**
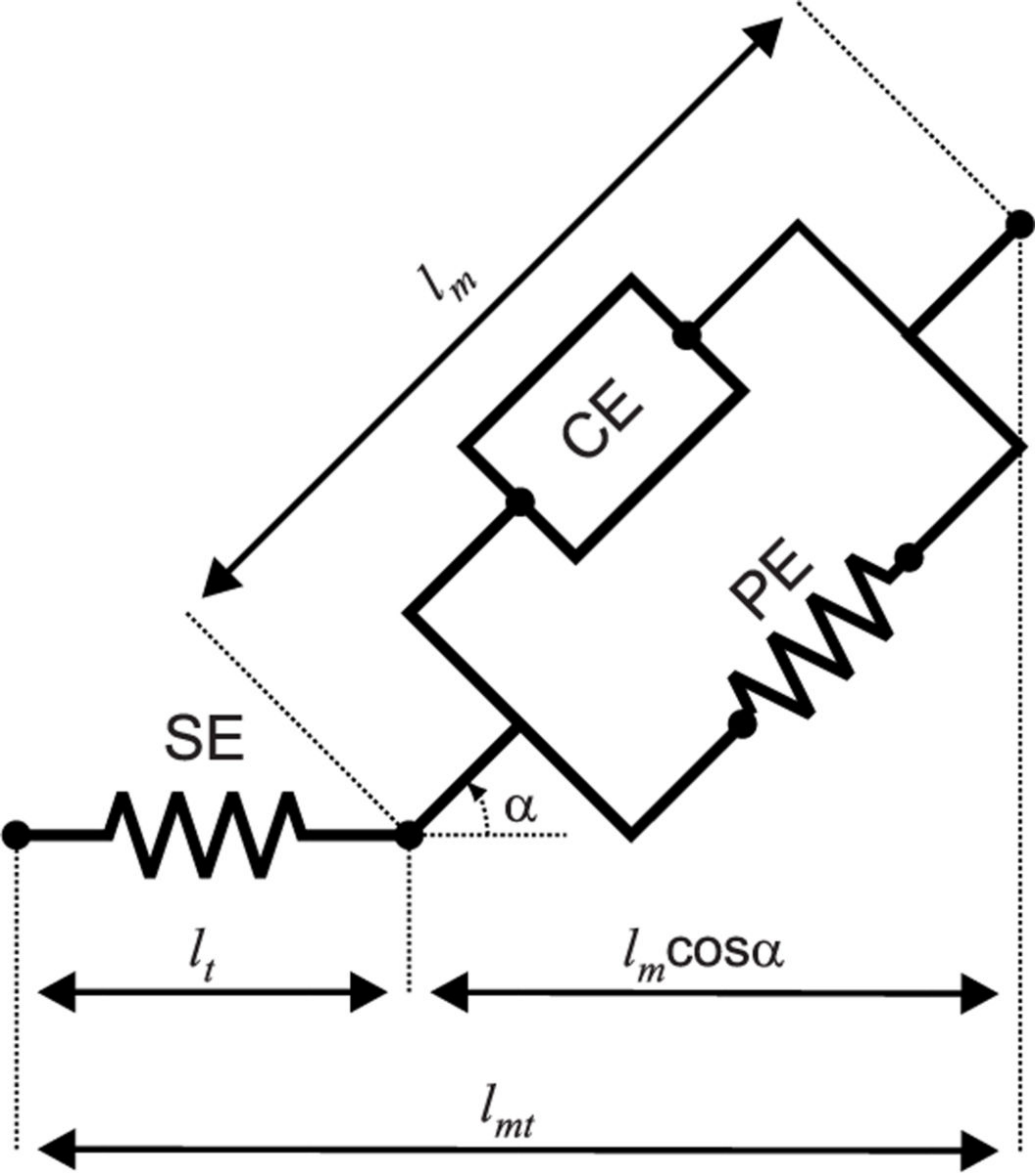
Hill-type muscle model describing the force generation by the muscles. The contractile element (CE) or the active element produces active contraction forces. The parallel element (PE) prevents the muscle from over stretching the muscle-tendon unit during normal operation. The series element (SE) represents the series elasticity of the muscle, including the muscle-tendon. Contractile element length or fiber-length ($l_{m}$) is the length of muscles fibers. Pennate muscles are defined by the pennation angle *α_o_*. Series tendon-length ($l_{t}$) is the length of series element. The total length of the muscle ($l_{mt}$) is defined as $l_{mt}=l_{m}\cos(\alpha )+l_{t}$.

Hill-type models make use of passive elements such as springs and dampers and experimental data to represent the active and passive dynamics of a muscle [46]. The contractile element (CE) and the parallel elastic element (PE) represent the muscle fibers; the series elastic element (SE) represents the total series elasticity of the muscle-tendon complex. It is important to keep in mind that due to this definition even muscles with short or no tendon still have non-zero tendon lengths in the parameterization of the Hill-type muscle model. Fig. 3 shows the formulation of the muscle model used in this work. To simplify the dynamics of the muscle model, we assumed rigid tendons [47].

The Hill-type muscle model illustrated in Fig. 3 was characterized by the following parameters: the optimal fiber length ($l_{m}^{o}$; the length of muscle-fiber at which the muscle produces maximal active force), the tendon slack length ($l_{t}^{s}$; the length of the tendon below which the muscle transfers no force to the attached bones), the maximum muscle-fiber velocity ($v_{m}^{max}$), the pennation angle when the fiber length is at its optimal ($\alpha_{o}$), and the maximum isometric force ($F_{m}^{0}$). The formal description of the Hill-type muscle model used [47] is described in Appendix A.

#### 2) MUSCLE ATTACHMENT POINTS

**FIGURE 4.**
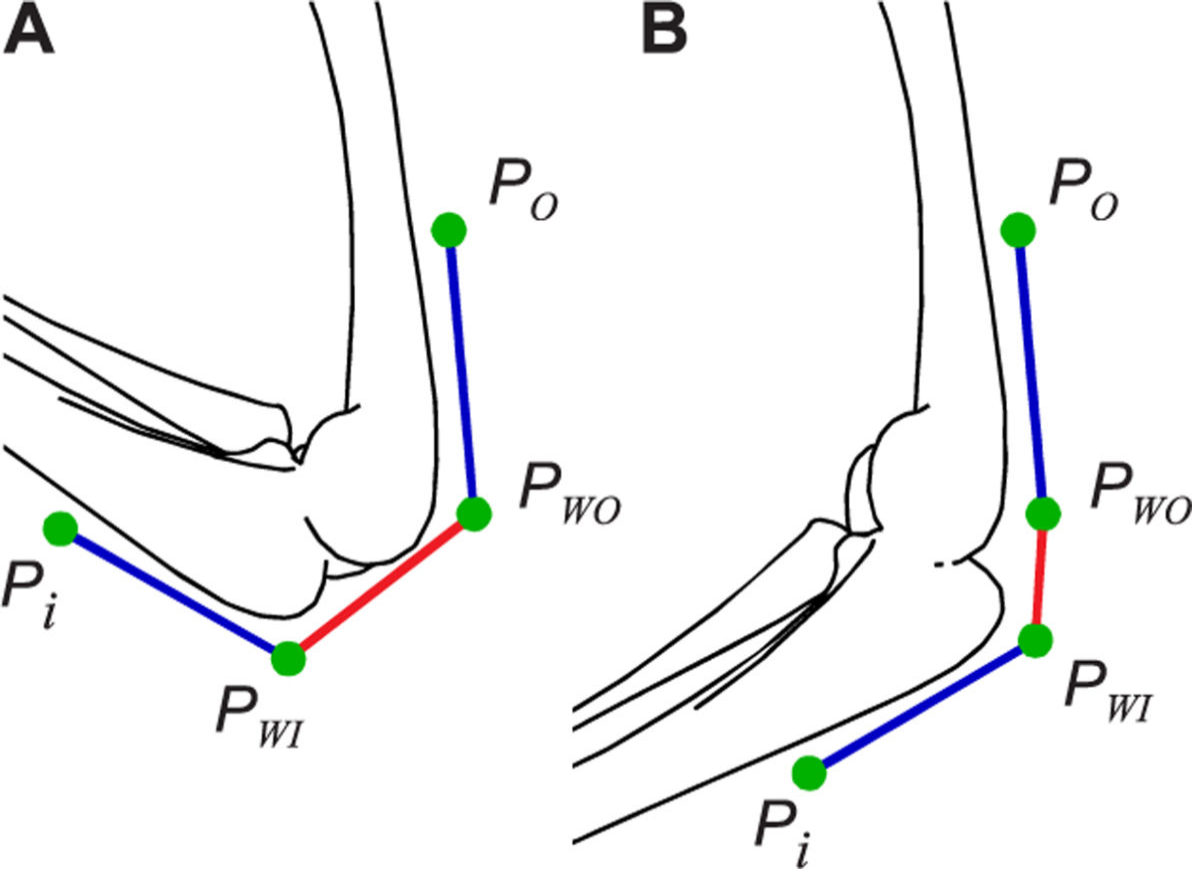
Polyline approximation of muscle paths. (A) and (B) show an example of an extensor muscle around the knee joint. Coordinates *P*_*o*_(origin) through *P*_*I*_(insertion) define the polyline muscle path. Coordinates *P*_*O*_ through *P*_*WO*_(waypoint) are attached to the femur and coordinates *P*_*WI*_(waypoint) through *P*_*I*_ are attached to the tibia. Thus, *P*_*WO*_ − *P*_*WI*_ is the only segment of the polyline that changes the muscle length when the joint is flexed or extended.

Muscles are attached to the bones via tendons. The attachment at the most proximal bone is called the origin (that bone tends to move less during muscle contraction) and the one at the most distal bone is called the insertion (that bone tends to move most during muscle contraction). The complex muscle paths were approximated as a polyline, a sequence of straight lines starting at the origin and ending with the insertion and are connected by *waypoints* in between (Fig. 4). The polyline approximation is a common approach to describe the muscle paths [18], [26], [34], [48]. (Refer to Fig. 4 for an example showing the polyline muscle path description around the knee joint). The identification of the muscle attachment points and waypoints for hind and forelimbs used in this study is described below.

##### a: HINDLIMB ATTACHMENT POINTS

**FIGURE 5.**
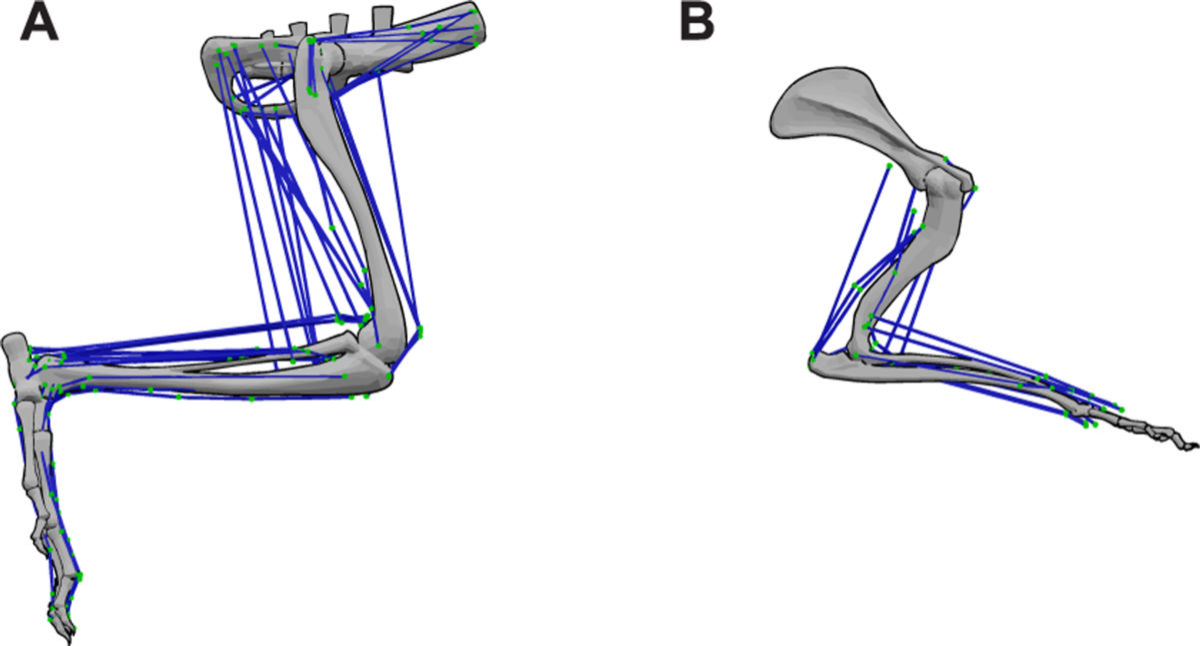
Lateral view of the musculoskeletal system of the mouse. (A) right hindlimb showing the attachment of 42 muscle-tendon units and (B) right forelimb showing the attachment of 17 muscle-tendon units. For all computations in this paper, the pose of the limbs shown in (A) and (B) are used unless mentioned otherwise.

The attachment points for mouse hindlimb muscles have been previously identified by Charles *et al.* [31], [49]. However, because of the differences in the bone geometry between our model and Charles model [31], it was necessary to transfer the muscle attachment points from Charles model to the bone surfaces of our mouse model. To automate the transfer process and limit the errors, we first performed mesh registration over the two bones from both models (mesh registration involves identifying appropriate landmarks between each bone segment of the hindlimb of our model and theirs). Based on the chosen landmarks, a coordinate transformation matrix was computed to describe an affine transformation from the bone mesh in Charles model to our model. The affine transformation obtained from the mesh registration was then used to transfer the attachment points and waypoints between the models. The above-described process was carried out using the open-source mesh software CloudCompare [50]. Identification of the landmarks on each bone from the two models was done manually. This process was carried out for the pelvis, femur, tibia, and pedal (tarsus, metatarsus, phalange) bones of the mouse hindlimb (Fig. 5A).

##### b: FORELIMB ATTACHMENT POINTS

Modeling the forelimb muscles was challenging because of the lack of prior studies identifying attachment points of forelimb muscles. Delaurier *et al.* [51] has previously developed a 3D forelimb atlas of the mouse embryo at embryonic day 14.5 using Optical Projection Tomography and digital segmentation. Although the model was based on data from a mouse embryo, it still provides useful information on muscle attachments to the bones. This data allowed us to identify attachment points and necessary waypoints for 17 forelimb muscles (Fig. 5B).

#### 3) ESTIMATION OF MUSCLE PROPERTIES

As described above, the Hill-type muscle model is characterized by four parameters. Of these, the fiber velocity $v_{m}^{max}$ is dependent on the fiber length and can be expressed as a function of optimal fiber length $l_{m}^{o}$. As in Charles *et al.* [52], we set $v_{m}^{\max}$ to 10 $l_{m}^{o}/\text{s}$. Thus, $l_{m}^{o}$, $l_{t}^{s}$, $\alpha_{o}$ and $F_{m}^{0}$ had to be identified for each muscle in the model.

##### a: HINDLIMB MUSCLE PROPERTIES

Transferring the attachment points from Charles’ model to our model meant that the muscle parameters had to be scaled accordingly. Maximum isometric force $\left(F_{m}^{0}\right)$ and pennation angle (*α_o_*) were retained to be the same as in Charles model and only the length dependent parameters [optimal fiber length $\left(l_{m}^{o}\right)$ and tendon slack length $\left(l_{t}^{s}\right)$] were scaled. Since the length of the muscle varies over the DoFs it spans, we computed a scaling factor that is satisfied at all feasible joint poses. To achieve this, we employed a numerical optimization algorithm based on the one proposed by Modenese *et al.* [53] to compute the scaling factors for each muscle. Refer to the Appendix B for further details.

##### b: FORELIMB MUSCLE PROPERTIES

Parameters $l_{m}^{o}$ and $F_{m}^{0}$ were extracted from measurements made by Mathewson *et al.* [54] and used as preliminary approximations. $l_{m}^{o}$ was scaled in proportion to the muscle-tendon lengths (i.e., the ratio of muscle-tendon length measured by Mathewson *et al.* [54] to the muscle-tendon length in our model for each forelimb muscle was used as the scaling factor for $l_{m}^{o}$). The scaling procedure used for hindlimb muscle parameters could not be employed here because there was no previous biomechanical model of the mouse forelimb. $F_{m}^{0}$ was computed by multiplying the physiological cross-sectional area (PCSA) of the muscle by isometric stress (*σ*) taken to be 0.3 N/mm^2^, the same used by Charles *et al.* [31] for the hindlimb muscles.

The remaining muscle property, $l_{t}^{s}$, was estimated using an adaptation of the numerical optimization technique formulated by Manal and Buchanan [55]. We removed the restriction that muscle fibers only operate in the ascending region of the force-length curve. We extended the algorithm to consider all the DoFs a muscle operates on while optimizing for $l_{t}^{s}$. Refer to the Appendix C for further details.

#### 4) COMPUTATION OF MUSCLE-TENDON LENGTH AND MOMENT-ARM

Muscle-tendon length ($l_{mt}$) is the distance from the origin of the muscle on the proximal bone to the insertion point on the distal bone. Based on the polyline approximation for describing the muscle paths (Fig. 4),

(1) $$l_{mt}=\sum_{n=0}^{N-1}\left\|P_{n+1}-P_{n}\right\|$$

where *P_n_* are the muscle attachment points on the polyline with*P*_0_ being the origin, *P_N_* the insertion point and waypoints in between $P_{i}:i=1\ldots (N-1)$.

Muscle moment-arms (*r*) were computed using the perturbation method described in Sherman *et al.* [56],

(2) $$r=\partial l_{mt}/\partial \theta$$

where *θ* is generalized coordinate representing the DoF of interest across which the muscle moment-arm is to be computed.

### D. ANALYSIS OF SENSITIVITY OF JOINT MOMENT-ARMS AND MOMENTS TO CHANGES IN MUSCLE PARAMETERS AND ATTACHMENT POINTS

Like any model, the mouse biomechanical model was developed based on several assumptions and simplifications. To highlight and study the influence of various characteristics of the biomechanical model on the overall motor behavior, we analyzed the sensitivity of joint moment-arms and moments to changes in muscle attachment points and model parameters.

For both analyses, we used the Sobol method [57], a variance-based global sensitivity method. The Sobol method decomposes the proportion of model output variance caused by each individual parameter [58]. This method also allows for the study of the inter-parameter effect on the model’s output variance, but we restricted our analysis to first-order indices (main effects). First-order indices “measure the direct contribution of each input factor to the output variance” [58]. A value of 1.0 for the first-order indices indicates that the parameter is solely responsible for all the variance in the models’ output, whereas a value of 0.0 represents no influence on the models’ output variance. We used the root-mean-square (RMS) value of the joint moment-arm or moment over the DoFs range-of-motion to represent the scalar value necessary to evaluate the sensitivities. The Sobol sensitivity analysis was performed using SALib v1.4 [59], an open source python library for the sensitivity analysis.

The polyline approximation of the muscle paths determines the computation of muscle-tendon length ($l_{mt}$) and thereby the moment-arm (2). For the polyline approximation, change in $l_{mt}$ can be reduced to just two attachment points of the segment that cross the DoF of interest (refer to Fig. 4 showing an example). Therefore, for the analysis of sensitivities to muscle attachments, we referred to the proximal attachment point of the segment as *P_WO_* and to the distal one as *P_WI_*. The sensitivities to these attachment points were computed by varying their 3D position within a range of ±0.5 mm in x, y, and z directions. With *P_WO_* and *P_WI_* we had six parameters (x, y, and z coordinates for each point) to study the sensitivity of the joint moment-arm. We limited our analysis to study how the 3D location of the two attachment points influence the joint moment-arms. As the total sum of the first-order indices is equal to 1.0, we reported the sum of the first-order indices along the individual 3D coordinates (x-y-z) for each attachment point. These values represent a direction-independent measure of moment or moment-arm sensitivity to the attachment points. Data on the individual sensitivities are available in the supplementary material.

Muscle force production depends not only on the geometric relationship defined by the muscle-path but also on the muscle dynamics. The Hill muscle dynamics are parameterized by four main parameters (as described in section II-C1). $F_{m}^{0}$ linearly affects the overall force produced by the muscle. $l_{m}^{o}$, $l_{t}^{s}$ and *α_o_* determine the active and passive forces produced by the muscle and consequently affect the joint moments. Sensitivity of the joint moments to the changes in muscle parameters were analyzed within a range defined by ±10% of their original parameter values.

## III. RESULTS

The parameters of all muscles in the model are specified in Table 2. Because of the differences in the available experimental data, muscle parameters for the hindlimb and forelimb muscles were obtained using different approaches. For hindlimb muscles, the maximum isometric force $F_{m}^{0}$ and pennation angle (*α_o_*) were directly taken from Charles *et al.* [31] while tendon slack length ($l_{t}^{s}$) and optimal fiber length ($l_{m}^{o}$) were scaled using the method described in [53] to account for the differences in model dimensions. For forelimb muscles, $F_{m}^{0}$ and $l_{m}^{o}$ were scaled from Mathewson *et al.* [54] while keeping *α_o_* the same. $l_{t}^{s}$ was optimized using a modified version of the algorithm originally described by Manal and Buchanan [55] (see section II-C3.b for details).

### A. MOMENT-ARM ANALYSIS FOR THE HINDLIMB MUSCLES

**FIGURE 6.**
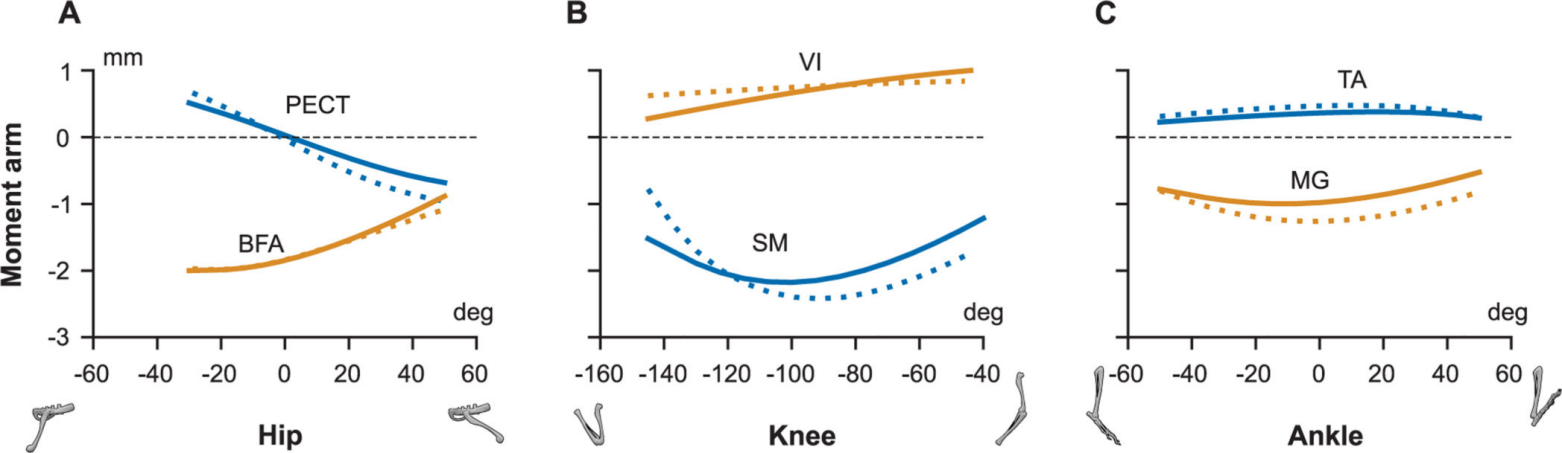
Comparison of moment-arms from [31] (dotted lines) and current model (solid lines) for muscles (A) *pectineus* (PECT) and *biceps femoris anterior* (BFA) over hip flexion-extension range-of-motion (RoM) (B) *semimembranosus* (SM) and *vastus intermedius* (VI) over knee flexion-extension RoM (C) *medial gastrocnemius* (MG) and *tibialis anterior* (TA) over ankle flexion-extension RoM. The moment-arms are normalized by the respective thigh length (thigh_charles_ = 16.25 mm and thigh_current_ = 24.5 mm).

Because of the differences in bone geometries, we transferred the muscle attachment points from the hindlimb model developed by Charles *et al.* [31], [49] to our model. The mesh registration technique described in section II-C2.a was used to transfer the attachment points. The attachment points of the muscle have a direct influence on the muscle moment-arms (Fig. 4) and consequently on the moments on the joints they influence. To compensate for the lack of wrapping surfaces, additional waypoints were introduced along the muscle path when necessary. To validate the muscle attachment process, moment-arms of two flexor muscles of the hip, knee, and ankle joints from Charles model were compared with the moment-arms in our model (Fig. 6). For this comparison, moment-arms were normalized to their respective thigh (femur) lengths, since the two models are of mice of different age and size. The moment-arms from the two models are in good quantitative and qualitative agreement throughout the range-of-motion for the three hindlimb joints.

### B. DESCRIPTION OF MUSCLE FUNCTION BASED ONMOMENT-ARMS AND MOMENTS

**FIGURE 7.**
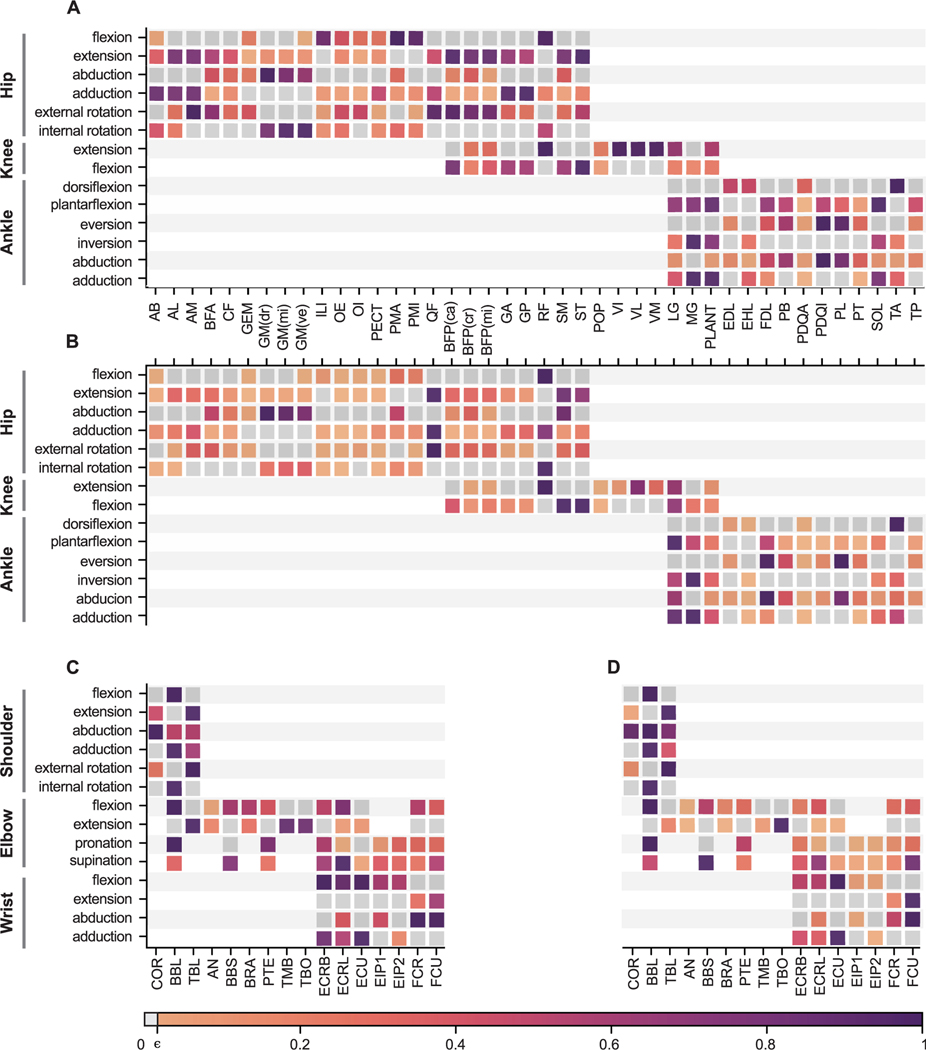
Maximum moment arms (A, C) and moments (B, D) for each muscle and joint function. Grey boxes indicate joint functions for joints the muscle spans but has zero influence over. For example, a pure hip flexor muscle is considered to be spanning over both hip flexion and extension joints, but the corresponding hip extension will be in grey to indicate that the muscle has no influence on hip extension. The moment-arms and moments are normalized by the muscle which has the maximum influence in the group. ϵ indicates a very low non-zero positive value.

Fig. 7 presents a comprehensive overview of each muscle’s influence over every DoF it spans. We present the functional grouping of the muscles based on the moment-arm and moment. Each DoF was sub-divided into two functions, representing the possible directions in which the muscle can influence the DoF. Thus, for every DoF it spans over, a muscle has the possibility to apply a moment either on one of the DoF functions’ or both (zero-crossing). Moment-arms were computed in the default pose shown in Fig. 5; moments were computed assuming maximal muscle activation (*a*(*t*) = 1.0).

#### 1) HINDLIMB MUSCLES

Fig. 7A,B shows the functional grouping of hindlimb muscles based on the moment-arm and moment, respectively. For hip flexion, *iliacus*, *psoas major*, *psoas minor*, and *rectus femoris* have the maximal moment-arms. However, since *rectus femoris* has the largest maximum isometric force, it exhibits the highest moment towards hip flexion. For hip extension, *adductor longus*, *adductor brevis*, *quadratus femoris*, *biceps femoris posterior*, *semimembranosus*, and *semitendinosus* have the dominant moment-arms. However, grouping them by moment reveals that *quadratus femoris*, *semimembranosus*, and *semitendinosus* are the dominant muscles. This classification also highlights that all hip flexors except *psoas major*, *psoas minor*, *iliacus*, and *rectus femoris* have zero-crossings (Fig. 7A). While *gluteus maximus* muscle has the highest moment-arm to abduct the hip, *semimembranosus* along with *gluteus maximus* also strongly contributes to hip abduction moments as their maximum force is larger. For hip adduction, *adductor brevis*, *adductor longus*, *adductor magnus*, *gracilis anterior*, and *gracilis posterior* are the dominant adductors when we consider the moment-arms. Considering moments, *quadratus femoris* and *rectus femoris* dominate over other muscles because of their large $F_{m}^{0}$. For hip external rotators *quadratus femoris* has the most significant moment-arm and moment in the group. Also, *gluteus maximus* has the largest moment and moment-arm among the hip internal rotators.

For knee flexion, *semitendinosus*, *biceps femoris posterior*, *gracilis anterior*, *gracilis posterior*, and *semimembranosus* have the strongest moment-arms, but *semimembranosus* and *semitendinosus* show the largest moments followed by *lateral gastrocnemius*. For knee extension, *rectus femoris*, *vastus intermedius*, *vastus lateralis*, and *vastus medialis* have the largest moment-arms, while *rectus femoris* exerted the largest moment, followed by *vastus lateralis* and *lateral gastrocnemius*.

Several muscles show strong moment-arms for ankle plantarflexion with *soleus* and *plantaris* having the largest moment-arms. Considering the moments, *lateral gastrocnemius* overshadows all the other ankle plantarflexors. For ankle dorsiflexion, *tibialis anterior* exhibits the strongest moment-arm and moment. Among ankle evertors, *peroneus digiti quarti* and *peroneus longus* show the largest moment-arm but *flexor digitorum longus* and *peroneus longus*, due to their large maximum isometric force, also significantly contribute to the moments. For ankle inversion, *medial gastrocnemius* has the highest moment-arm and moment among all the muscles in its group. *Peroneus digiti quarti*, *peroneus longus*, and *peroneus brevis* have large moment-arms for ankle abduction, while *lateral gastrocnemius*, *flexor digitorum longus*, and *peroneus longus* Can exert large moments for ankle abduction. *Medial gastrocnemius*, *plantaris*, and *soleus* have significant moment-arms for ankle adduction group, and *lateral gastrocnemius* and *medial gastrocnemius* have the highest moments.

#### 2) FORELIMB MUSCLES

Figs 7C,D show the functional grouping of forelimb muscles based on the moment-arm and moment, respectively. Anatomically, the forelimb is more complex than the hindlimb, the scapula is a floating link with all six DoFs and elbow joints have complex joint rotations. The kinematic complexities are reflected in the identification of muscle functions. Most shoulder joint muscles originate from vertebral processes. Here, we have not included those muscles that originate from the spinal segment due to the lack of available experimental data; the model only includes a limited number of proximal muscles in the forelimb. Shoulder retraction is actuated by the long head of *biceps brachii* whereas shoulder protraction is actuated by *coracobrachialis* and the lateral head of *triceps brachii*. Both latter muscles have similar maximum moment-arms, but the maximal isometric force of the lateral head of *triceps brachii* is much larger compared to *coracobrachialis*, resulting in a larger moment. *Coracobrachialis*, the long head of *biceps brachii*, and the lateral head of *triceps brachii* contribute similarly to shoulder abduction both in terms of moment-arm and moments, while the long head of *biceps brachii* and the lateral head of *triceps brachii* contribute to shoulder adduction. This shows that shoulder adduction is possible in the model only due to the zero-crossing of the long head of *biceps brachii* and the lateral head of *triceps brachii*. For shoulder external rotation, *coracobrachialis* and the lateral head of *triceps brachii* act on the joint function with the lateral head of *triceps brachii* dominating the joint function both in moment-arm and torque magnitudes. The long head of *biceps brachii* alone acts on the shoulder-internal-rotation joint function, leaving us to draw no further inferences about this joint function.

Among the many forelimbs muscles that span over the elbow joint, the elbow-flexors, the long head of *biceps brachii* and *extensor carpi radialis longus* have the largest moment-arms followed by short head of *biceps brachii*, *brachialis*, *extensor carpir adialis brevis*, and *flexor carpir adialis*. When we consider the moments, long head of *biceps brachii* has the largest maximal moment followed by short head of *biceps brachii*. Other muscles that have a large moment-arm are weak and exert smaller moments. For elbow-extension, all the triceps muscles (*triceps brachii lateral*, *triceps brachii medial*, and *triceps brachii long head*) have relatively strong moment-arms, with the long head of *triceps brachii* exerting the largest maximal moment. In the current model, we did not include any elbow-pronator muscles. For elbow supination, *extensor carpi radialis longus* has the largest moment-arm, followed by short head of *biceps brachii*, *extensor carpi radialis brevis*, and *flexor carpi ulnaris*. The short head of *biceps brachii* becomes the most dominant muscle for elbow supination when we consider moments, followed by *extensor carpi radialis longus* and *flexor carpi ulnaris*.

All the wrist flexor muscles have similar maximum moment-arms across the joint function, with *extensor carpi radialis brevis*, *extensor carpi radialis longus*, and *extensor carpi ulnaris* having the most dominant moment-arms. For moments, *extensor carpi ulnaris* has the strongest moment followed by *extensor carpi radialis longus* and *extensor carpi radialis brevis*. Three muscles act on wrist-flexion, of which *peroneus longus* has the highest moment-arm followed by *flexor carpi ulnaris*; when we consider moments, however, the dominance is flipped. While *flexor carpi radialis*, *flexor carpi ulnaris*, and *peroneus longus* have the strongest moment-arms for wrist abduction, the strongest moments are exerted by the *flexor carpis ulnaris*, followed by *flexor carpi radialis*. Among the three muscles that influence wrist adduction, *extensor carpi ulnaris* has the strongest moment-arm followed by *extensor carpi radialis brevis* and *extensor carpi radialis longus*. *Extensor carpi radialis longus* has a zero-crossing such that it influences both wrist abduction and adduction. For moments, *extensor carpi ulnaris* has the highest moment followed by *extensor carpi radialis brevis*.

### C. OPERATIONAL RANGE OF MUSCLE-FIBER LENGTH

**FIGURE 8.**
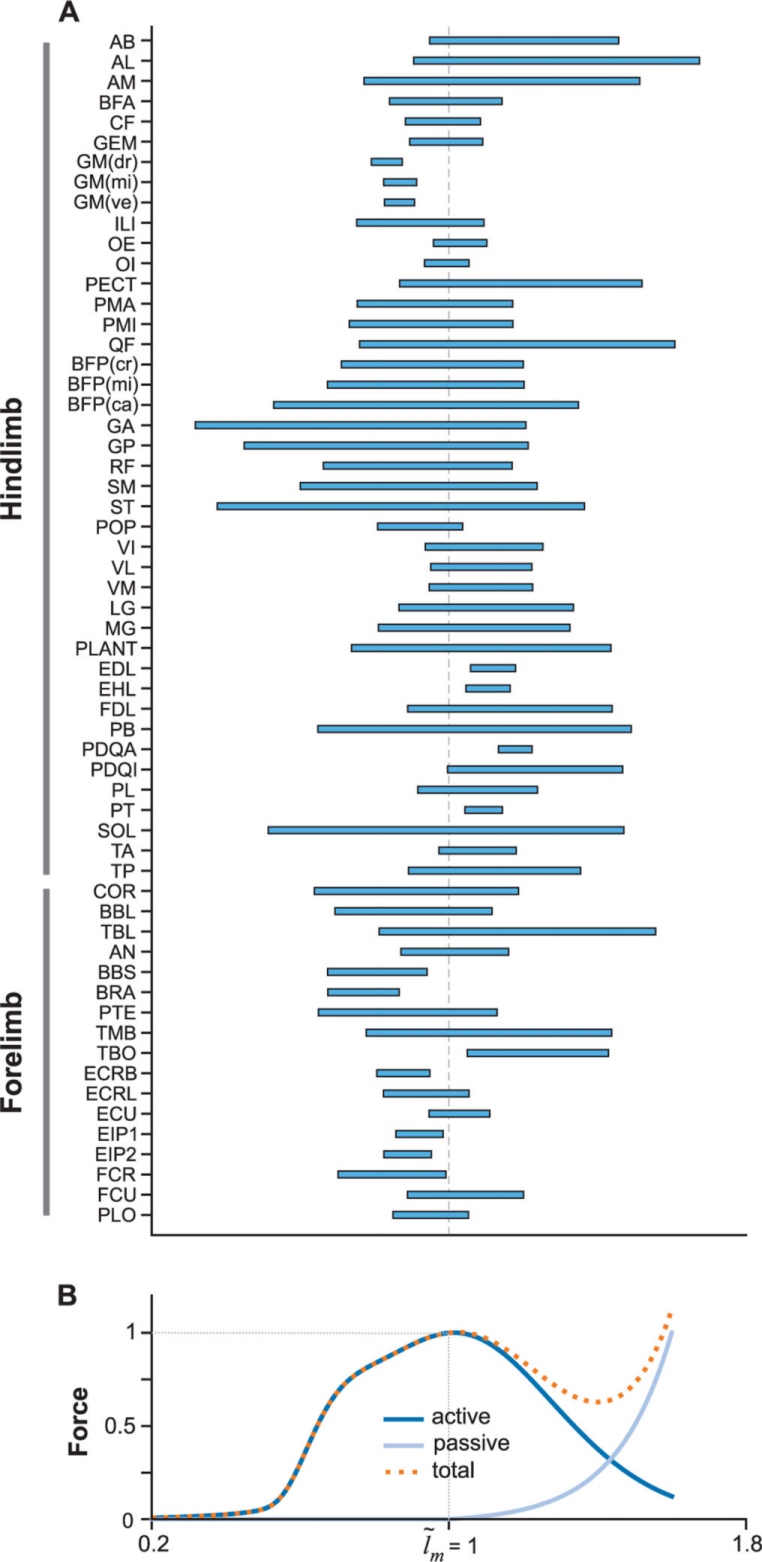
(A) Range of normalized muscle-fiber length (muscle-fiber length ($l_{m}$) normalized by optimal fiber length ($l_{m}^{o}$); ${\tilde{l}}_{m}=l_{m}/l_{m}^{o}$ for each muscle in the forelimb and the hindlimb. The range of ${\tilde{l}}_{m}$ is computed considering the range-of-motion of all the degrees-of-freedom the muscle spans. (B) Hill-type muscle force-length relationship showing the normalized force produced by muscle contraction (active force), by series and parallel elastic forces, and the sum of both (total force) across the ${\tilde{l}}_{m}$. At ${\tilde{l}}_{m}=1.0$ the muscle produces maximum active force in the force length curve.

The muscle-fiber length (*l_m_*) determines the working region of the muscle in the force-length (FL) curve (Fig. 8B). More specifically, the fiber-length determines if the muscle produces force purely based on muscle contractions or a combination of muscle contraction and passive forces. The range of operation in the FL relationship is determined by the muscle attachment points and parameters. In Fig. 8A we show the working ranges of normalized muscle-fiber lengths (${\tilde{l}}_{m}$) across all the DoFs each muscle spans. Muscles that span over a single joint tend to have shorter range of operation in the FL relationship. Most muscles in the mouse forelimb and hindlimb operate within the active (${\tilde{l}}_{m}\leq 1.0$) and passive force (${\tilde{l}}_{m}>1.0$) regions of the FL curve. *Gluteus maximus* in the hindlimb is the only muscle that operates completely in the active region of the FL curve. Short head of *biceps brachii*, *brachialis*, *extensor carpi radialis*, and *extensor indicis proprius* muscles in the forelimb exhibit operation only in the active region. These muscles in the hindlimb and forelimb have no passive/elastic force contributions during movement. In the hindlimb, *extensor digitorum longus*, *extensor hallucis longus*, *peroneus digiti quarti*, and *peroneus tertius* are the muscles that operate only in the passive region of the FL curve. All the mentioned muscles span over the ankle joint. *Pronator quadratus* is the only muscle in the forelimb that operates only in the passive region of the FL curve. This is because the optimization of the parameters of forelimb muscles was performed under the constraint that every muscle must have some operation range in the active section of the FL curve. A muscle whose operational range is only in the passive region always produces an elastic force on the joints and the force increases exponentially as the muscle is stretched. Passive forces are useful as they consume no energy to produce movement but if the desired movement is against the passive force, then it requires additional energy to overcome it. Hence, there is an interesting optimum that could be reached to minimize energy consumption. All the other muscles in the hindlimb and forelimb operate across the active and passive regions depending on the pose/configuration of the joints.

### D. SENSITIVITY ANALYSIS

Muscle attachments and muscle parameters together determine the overall function and moments produced by the muscle. The high number of muscles and the DoFs each muscle influences makes it a challenging task to perform a comprehensive sensitivity analysis. Here, we use the Sobol method, a variance-based global sensitivity method to perform the analysis (see Section II-D).

**FIGURE 9.**
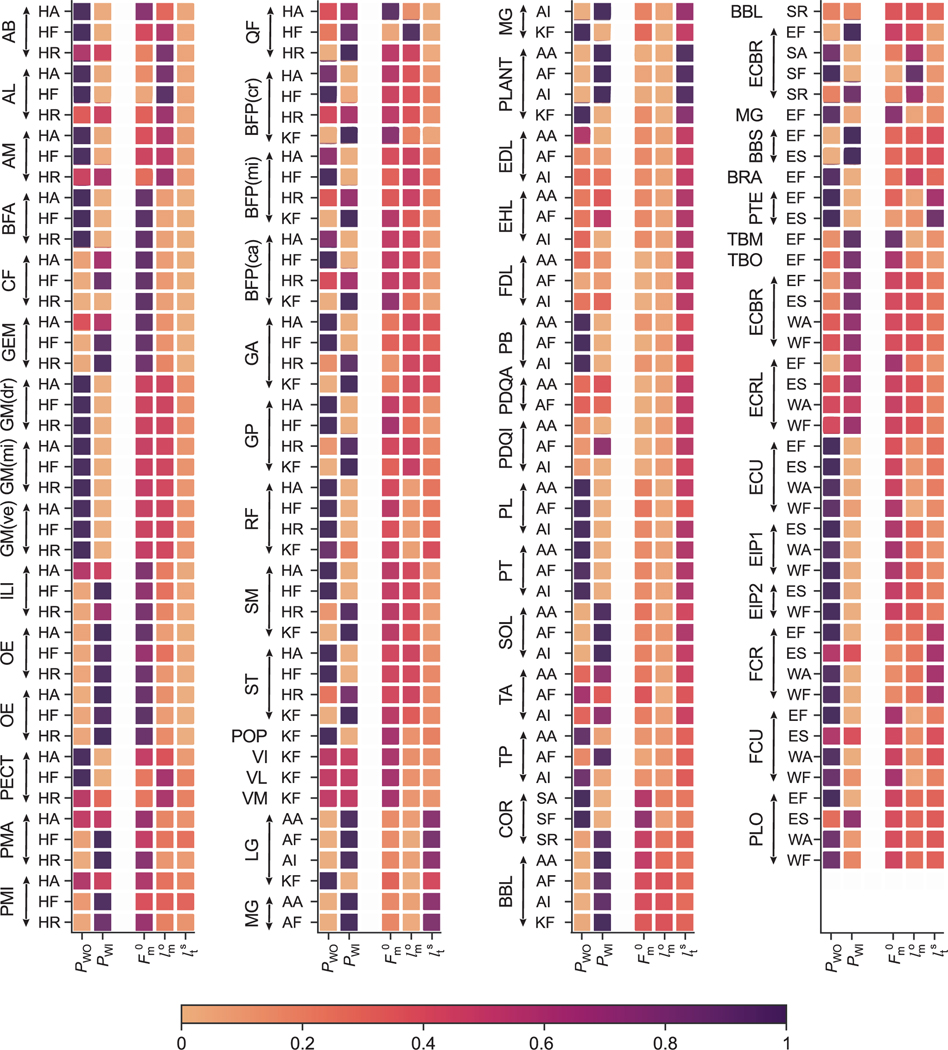
Sensitivity of joint moment-arms to changes in muscle attachment points and of joint moments to changes in muscle parameters. Analysis of muscle attachments and muscle parameters was done independently but is shown together in the figure. The colors indicate the first-order indices from the Sobol analysis. A first-order index value of 0.0 indicates that the parameter under observation has no contribution to the output’s (moment-arm/moment) variance and value of 1.0 indicates that the parameter is responsible for the total output’s variance. Analyzed DoF pairs are: Hip abduction-adduction (HA), flexion-extension (HF), internal-external rotation (HR), Knee flexion-extension (KF), Ankle abduction-adduction (AA), flexion-extension (AF), inversion-eversion (AI). Shoulder abduction-adduction (SA), flexion-extension (SF), internal-external rotation (SR), Elbow flexion-extension (EF), pronation-supination (ES), Wrist abduction-adduction (WA), flexion-extension (WF).

It is important to note that in Fig. 9, results of two separate sensitivity analyses are displayed, one for the muscle attachments and one for the muscle parameters. Thus, sensitivity indices for the two cases should be interpreted independently.

#### 1) SENSITIVITY OF JOINT MOMENT-ARMS TO CHANGES IN MUSCLE ATTACHMENT POINTS

**FIGURE 10.**
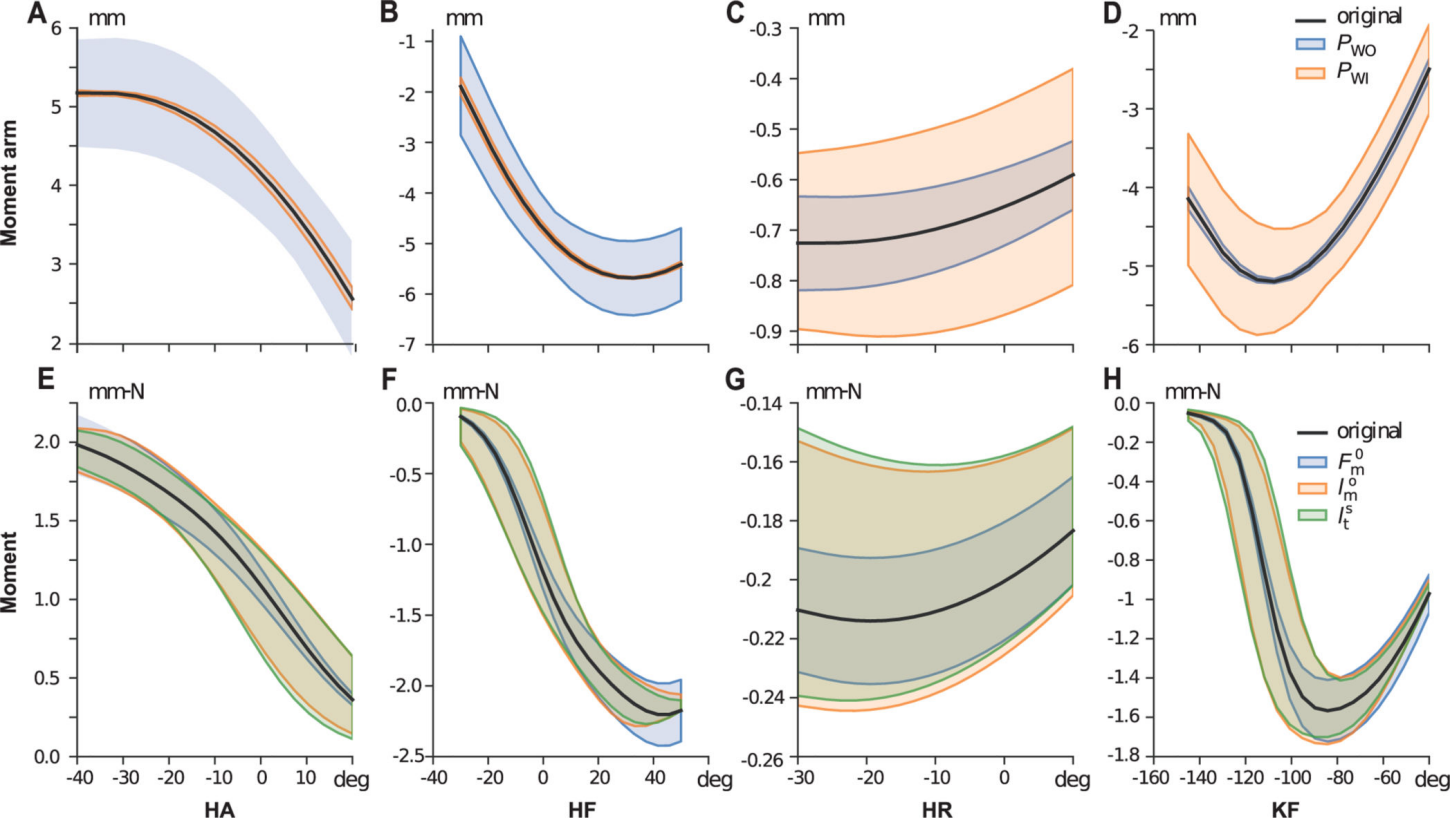
(A-D) Variation of *gracilis anterior* moment-arms for the variation of the 3D position of the attachment points *P*_*WO*_ (attachment point in the parent bone of the joint) and *P*_*WI*_ (attachment point in the child bone of the joint) within the range of 0.5 mm for hip flexion-extension (HF), hip abduction-adduction (HA), internal and external rotation (HR) and knee flexion-extension (KF). (E-H) Variation of Gracilis anterior moments for the variation of the muscle parameters (maximum isometric force ($F_{m}^{0}$), muscle fiber length ($l_{m}$), tendon slack length ($l_{t}$) within the range of 10% of their default values for HF, HA, HR and KF.

The first observation we can make from the sensitivity analysis to variation in attachment points is that the majority of muscle joint moment-arms are highly sensitive to either *P_WO_* (attachment to the parent bone of the joint) or *P_WI_* (attachment to the child bone of the joint) and only very few to both. Fig. 10(A-D) shows an example of the variation of moment-arms when the 3D positions of *P_WO_* and *P_WI_* are varied between ±0.5 mm for gracilis anterior muscle across the 4 DoFs it spans over the hip and the knee joints. For hip flexion-extension and abduction-adduction, moment-arms of *gracilis anterior* are highly sensitive to *P_WO_*. For hip internal and external rotation and knee flexion-extension DoF, the moment-arm of *gracilis anterior* is more sensitive to changes in *P_WI_* than *P_WO_*. The same observations are reflected in the comprehensive representation shown in Fig. 9.

Referring to Fig. 4, we can interpret the geometric reason for why a muscle-joint pair is sensitive to either *P_WO_*, *P_WI_*, or sometimes both. When the attachment points are perturbed to perform the sensitivity analysis, points further away from the joint rotation centers will result in larger changes in muscle length within the range-of-motion of the joint. As we have seen from (2), larger change in muscle-tendon length for the same change in DoF motion results in larger moment-arms. Thus, the proximity of the attachment point to the joint’s center-of-rotation will influence its sensitivity. From the analysis (Fig. 10), we can identify those attachment points that are most important and interesting to explore for a given muscle-DoF pair.

#### 2) SENSITIVITY OF JOINT MOMENT TO CHANGES IN MUSCLE PARAMETERS

In addition to the muscle attachment points, the muscle parameters determine the dynamics of the muscle and the moments it generates on the joints. Estimation of muscle parameters is a challenging process that could potentially lead to modeling errors. This applies to our forelimb model, where the muscle parameters were estimated based on several different data sources (See Section II-C3.b). The sensitivity analysis provides an overview of the parameters that have most influence on the moment generation for each muscle-joint pair. We performed this analysis for four muscle parameters [maximum isometric force $F_{m}^{0}$, optimal fiber length ($l_{m}^{o}$), tendon slack length ($l_{t}^{s}$), and pennation angle (*α_o_*)] within a range of ±10% of their original values. The sensitivities of joint moments to changes in muscle parameters are reported in Fig. 9. We do not show the results for *α_o_*, since no joint moment exhibited a significant sensitivity.

From the Hill-type muscle force (see (3) in the Appendix) $F_{m}^{0}$ has a linear influence on the muscle force while $l_{m}^{o}$ and $l_{t}^{s}$ have complex, non-linear relationships.

Among the hindlimb muscles that span the hip joint, $F_{m}^{0}$ is the parameter influencing the joint moments the most; $l_{m}^{o}$ being the next parameter in a few muscles in this group. No muscle in this group exhibits sensitivity for the choice of $l_{t}^{s}$ parameter. Next, sensitivity of moments caused by muscles that span hip and knee joints are equal due to the changes in $F_{m}^{0}$, $l_{m}^{o}$ and sometimes $l_{t}^{s}$. Muscles spanning only the knee joint exhibit sensitivity only for $F_{m}^{0}$. Moments of muscles that span knee and ankle or only ankle joint all have $l_{t}^{s}$ as their most important parameter. Among the forelimb muscles, moments are most sensitive to the $F_{m}^{0}$ parameter for almost all muscle-joint pairs. With just a few muscles for which moments are more sensitive to $l_{m}^{o}$ or $l_{t}^{s}$ parameters.

## IV. DISCUSSION

We presented a whole-body three dimensional (3D) musculoskeletal model of the mouse with a fully articulated skeletal system actuated by identified musculature for both hindlimbs and forelimbs. Using the model, we performed a systematic and comprehensive analysis of the limb musculature to study their influence on limb joints. We first studied how the muscles influence joint function based on the moment-arm and moments they exert. The analysis gives a comprehensive view to characterize muscle function. Our results reveal that many muscles that span multiple degrees-of-freedom (DoFs) tend to have zero-crossing (i.e., change their function over the DoFs range-of-motion). Examining the muscle-fiber length range showed how the limb muscles distribute their force production in terms of active and passive forces over the joints’ complete range-of-motion. We then performed a sensitivity analysis to highlight the crucial parameters in the model and showed how different parameters affect on each muscle-DoF pair in the model. Although the model was based on a number of simplifications and assumptions, it is an important step in the direction of building complex biomechanical, and ultimately neuromechanical models to study motor behaviors and their underlying neuronal control.

### A. MUSCLE SYSTEM DEVELOPMENT

Identification and characterization of the muscles operating in complex musculoskeletal systems is a challenging task. It involves a laborious process of extracting individual muscles from the animal, carefully identifying the attachment landmarks such as origin and insertion locations of each muscle and identification of major muscle parameters.

These steps were traditionally performed directly on cadavers [35]; later studies, used microCT scans along with digital segmentation, which lead to more detailed identification about muscle geometry and attachments, especially for deep muscles [49]. In this work, we developed the muscle system of both hindlimb and forelimbs by incorporating data from several studies on the mouse.

#### 1) TRANSFERRING HINDLIMB MUSCLE ATTACHMENT POINTS FROM CHARLES MODEL

Development of the hindlimb musculature was largely based on the OpenSim single mouse hindlimb model of Charles *et al.* [31], [49]. Since, this model was only of a single hindlimb, we had to transfer the muscle system to our full mouse model which had a different bone geometry. The first step in this process was to identify the appropriate landmarks of origin and insertion points of each muscle on the new model. To accomplish this task, we setup an automatic process to identify a coordinate transformation between the bones of two models using mesh-registration technique. With this, every attachment point of amuscle defined in a particular coordinate of a bone was transferred to the coordinate frame of the same bone in the current model. The model in Opensim had incorporated muscle wrapping surfaces to better describe the muscle paths. However, in our current framework, muscle paths were approximated as linear polyline paths similar to [18], [26], [48]. To compensate for this approximation, we had to manually introduce additional waypoints to describe the muscle path closer to the original model. With the use of polyline method it was possible to faithfully describe muscle paths. In figure 6, we compared moment-arms of six hindlimb muscles from our current model with the model developed by Charles *et al.* in OpenSim. Among the six muscles, five of them had used wrapping surfaces in Charles model (except *tibialis anterior* (TA)). The comparison showed excellent qualitative and quantitative agreement between the moment-arms of the two models, highlighting that the approximation of polyline method captured muscle paths well throughout range-of-motion.

#### 2) SCALING OF HINDLIMB MUSCLE PARAMETERS

After transferring the muscle attachments from Charles’ model to our model, it was necessary to scale the muscle parameters appropriately to our model’s geometry. Since our model is a whole-body 3D model, it was very important to consider the influence of the muscle parameters on all the DoFs the muscle spans. We used the numerical optimization based algorithm proposed by Modenese *et al.* [53] to appropriately scale $l_{m}^{o}$ and $l_{t}^{s}$ parameters considering all the DoFs a muscle spans. While the method emphasizes on scaling the parameters such that the change in muscle length was preserved between the original and the scaled model, there is no constraint on preserving the ratios between $l_{m}^{o}$ and $l_{t}^{s}$. In Charles model, $l_{t}^{s}$ was estimated based on the numerical method proposed by Manal and Buchanan [55]. Thus, the $l_{t}^{s}$ parameter has no direct measurable tendon property of the muscle. This is in accordance with the original formulation of the Hill-type muscle models [46]. Also, Charles *et al.* showed that the measured tendon lengths were either longer or shorter than the estimated $l_{t}^{s}$ values [31]. Because of these observations, we did not impose any constraints to the algorithm to preserve the ratios between $l_{m}^{o}$ and $l_{t}^{s}$ of Charles’ model. During future iterations of the model improvement, $l_{t}^{s}$ parameter could be estimated from animal experiments [22].

#### 3) MODELING THE MOUSE FORELIMB IS MORE CHALLENGING THAN THE HINDLIMB

Developing the forelimb muscle system was more challenging due to the lack of available biomechanical studies of the mouse or even rat forelimbs. To the best of our knowledge, Mathewson *et al.* [54] was the only published work that measured some of the muscle properties necessary to model Hill-type muscles. But, since the goal of their work was not building a simulation model of the mouse forelimb, information about muscle attachments were not reported.

Delaurier *et al.* [51] developed a 3D model of mouse embryonic forelimb using Optical Projection Tomography and digital segmentation. We transferred the attachment points for each muscle using their 3D atlas to our model. The choice of muscle was based on muscle data reported in [54]. This limited the forelimb model to mostly distal muscles. Proximal muscles around the shoulder that had origins from the spine were omitted from the model.

Mathewson *et al.* [54] reported $l_{m}^{o}$ and *α*_0_ for the forelimb muscles. Unlike in the hindlimb case, we could not employ the algorithm to scale length related parameters from Modenese *et al.* [53] because of a lack of information about muscle lengths at different model poses. The $l_{m}^{o}$ parameter was thus scaled based on the ratio of average muscle-tendon length for while *α*_0_ was used as reported. $F_{m}^{0}$ was computed as by multiplying the physiological cross-sectional area by the same value of maximum isometric stress used for hindlimb muscles (0.3 N/mm^2^).

The final missing parameter $l_{t}^{s}$ was estimated based on the extended algorithm by Manal and Buchanan [55]. (Refer to Appendix C for details on the changes incorporated to the algorithm.) Determining $l_{t}^{s}$ is one of the biggest bottlenecks in Hill-type muscle parameterization as there is no method to experimentally to estimate it [31]. The closest experimental method to estimate $l_{t}^{s}$ is described by Cox *et al.* [22]. However, performing such experiments are extremely difficult in the mouse because of their relatively small size. The modifications we proposed to the numerical method by Manal and Buchanan [55] should improve the reliability of these estimates for the mouse as well as for other musculoskeletal model of animals.

To the best of our knowledge there is no published work that characterizes either the muscle properties or muscle attachments for forelimb muscles attaching to the scapula or the spine; neither for mice nor rats. Hence, these muscles were considered beyond the scope of this work.

### B. MUSCLE MOMENT-ARMS AND MOMENTS

The model includes 59 distinct forelimb and hindlimb muscles. It is a challenging task to provide a comprehensive analysis of a complex model such as this. Conventionally, the practice is to report and describe the relationship between moment-arm and joint movement for each muscle individually. This means that it is necessary to assume the function of a muscle *a priori*. Instead of making this assumption, we reported a global view of the possible roles of a muscle based on moment-arm and moment (Fig. 7). With this representation one can quickly identify the function of a muscle and observe the contribution of different muscles to a particular degree of freedom.

**FIGURE 11.**
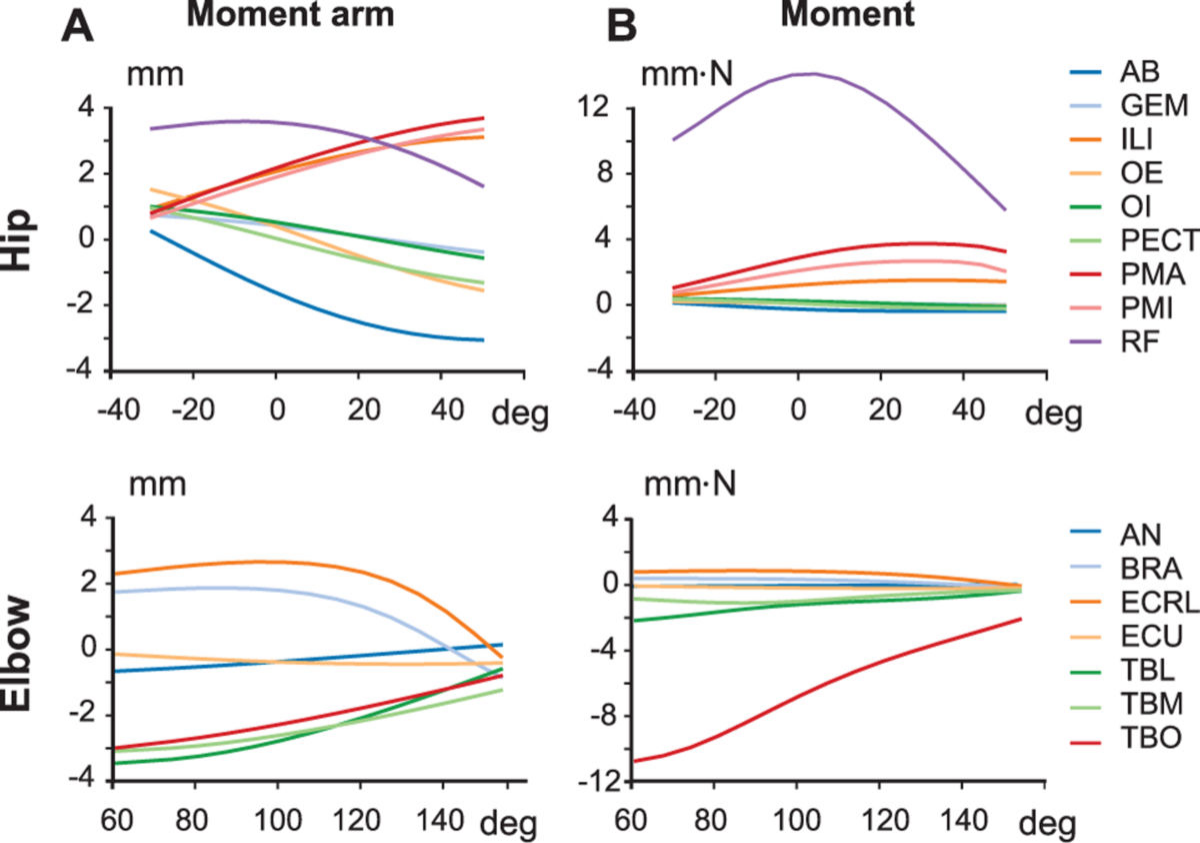
(A) Moment and (B) moment-arm of hip flexor muscles *adductor brevis* (AB), *gemellus* (GEM), *iliacus* (ILI), *obturator externus* (OE), *obturator internus* (OI), *pectinus* (PECT), *psoas major* (PMA), *psoas minor* (PMI), *rectus femoris* (RF). (C) Moment and (D) moment-arm of elbow flexor muscles *anconeus* (AN), *brachialis* (BRA), *extensor carpi radialis longus* (ECRL), *extensor carpi ulnaris* (ECU), *triceps brachii lateral head* (TBL), *triceps brachii medial head* (TBM), *triceps brachii long head* (TBO).

Previous musculoskeletal modeling studies have reported the behavior of zero-crossing of muscle moment arms in several animals such as cat [61], mouse [31], rat [35] and ostrich [23]. Young *et al.* [61] speculated that muscles with a zero-crossing moment-arms could intrinsically stabilize the joints around which they change sign without any need for extra neural commands. For the mouse hindlimb model [31] several muscles were reported to have a zero-crossing. But their analysis was limited to the assumption of functional roles assigned to each muscle *a priori*. Here, we identified more muscles that have zero-crossings. For example, previously only *pectineus* muscle was reported to have a zero-crossing for hip flexion-extension. From Fig. 11A,B we can observe that in addition to *pectineus* we have *adductor brevis*, *gemellus*, *gluteus maximus* (ventral), *obturator externus* and *internus* and *quadratus femoris* muscles have zero-crossing. Similarly for the different joints in forelimb and hindlimb, we can observe many muscles exhibiting the zero-crossing behavior.

A shortcoming of the representation we proposed is that it does not show joints’ angle information: it is not possible to see at what joint angle the zero-crossing occurs. This limitation is only in the visual representation in this article, and detailed moment-arms of all the possible muscle and joint combinations are available. We encourage the readers to use the data and plotting tools in the code-repository (see section V) to extract the detailed plots.

Along with moment-arms, we also reported the muscle moments across each DoF (Fig. 7). By presenting both moment-arms and moments together, we can quickly observe how the role and importance of a muscle in actuating a particular DoF can change. Muscles with large maximum isometric force naturally become the dominating muscle for the DoF. While the moment-arm is only dependent on the muscle attachments and joint position, moments also depend on the dynamics of the muscle—most importantly muscle activation. From a neural control point of view this is very interesting: the nervous system can operate with a single strong muscle and/or utilize several different weaker muscles together to produce the same movement. Co-activation of multiple synergistic muscles with respect to a specific function could also result in a stabilization of the joint in the other DoFs.

The current muscle moment-arm and moment results were computed while the joint of interest was rotated within the range of motion and while keeping all other joints in their default position. This has a strong influence on the results. It limits the scope of the analysis to a particular pose of the model. This is often the case in biomechanical model analysis as it becomes very complex to interpret and represent the relationship of different joints with muscle moment and moment-arm. Young and colleagues [26], [61] studied the coupled effect of a bi-articular muscle on joints. Also, we see from Fig. 7 that many muscles operate on more than two degrees of freedom, especially muscles that span joints with multiple degrees of freedom (e.g., hip, ankle, shoulder, or wrist). With the condensed representation (Fig. 7), it is possible to generate plots to study the relationships at various model poses.

### C. RANGE OF NORMALIZED MUSCLE-FIBER LENGTHS

Muscle-fiber length ($l_{m}$) is a state variable in describing the muscle dynamics (see Appendix A). It depends on the muscle-tendon length ($l_{mt}$), the muscle contraction dynamics, and the muscle activation. $l_{m}$ is thus a very interesting variable to study. In this paper, we reported the operational range of normalized muscle-fiber length (${\tilde{l}}_{m}$) for each muscle over all the joints it spans in Fig. 8. (Unlike the moment-arm and moment representation in Fig. 7, Fig. 8 incorporates all the possible joint poses for a single muscle.) The range of ${\tilde{l}}_{m}$ reflects the choice of the tendon slack length $l_{t}^{s}$ and optimal fiber length ($l_{m}^{o}$) parameters. In future, if we can obtain experimental data of fiber-lengths at the different limb postures. This data can be used to validate the model by making sure that the experimental measurements fall within the predicted ranges of ${\tilde{l}}_{m}$.

Analyzing the ${\tilde{l}}_{m}$ is also useful in understanding the behaviors by being able to characterize if a muscle is operating in the ascending, plateau or the descending region in the force-length curve (Fig. 7B). In smaller animals, such as mice, due to their low weight and small inertias, the effect of gravity on the body is negligible [62], [63]. With lower influence of gravity, the forces produced by the passive elements become more significant. This has two important consequences on the neural control circuits. One, it is less essential (compared to larger vertebrates) for the neural system to monitor the direction of gravity on the limbs during movements. Second, the momentum of the limbs is less useful during the swing phase during locomotion. For further discussion on the influence of body size on neural control refer to Hooper [62]. Thus, passive forces in mice play an important role in movement generation and it would be informative to use the muscle-fiber length range plots to explore it.

In [64], the authors reported the ${\tilde{l}}_{m}$ of human leg muscles during different speeds of walking and running. They observed that the ${\tilde{l}}_{m}$ has a wide range of operation in the force-length curve for different speeds of walking and running. Fig. 8 for the mouse model also highlights that most muscles with the current muscle parameters operate both in the ascending and descending region of the force-length allowing for the kind of variability observed in humans.

### D. SENSITIVITY ANALYSIS

While developing a complex model like the one described here, it is important to identify the critical parameters that influence the overall performance of the model. To this aim, we performed a variance based global sensitivity analysis using the Sobol method to systematically study the influences of muscle attachment points and muscle parameters on the moment-arms and moments.

In our analysis of sensitivity of moment-arms to muscle attachment points, we considered only those attachment points (*P_WO_* and *P_WI_* ) that influence the muscle-length effect on the joint of interest. In Charles *et al.* [31], the analysis was performed directly at the origin and insertion points, and the perturbations applied to the attachments were along a particular direction. In contrast, in our work, we applied perturbations to the attachments in all three directions. Similar to the observations made by Charles *et al.* in [31], we unsurprisingly observed that the attachment points located farther from the joint rotation centers had the greatest effect on the joint moment-arms. With the polyline approximation it therefore becomes very important to identify the intermediate points as accurately as possible to describe the muscle paths.

We further analyzed the sensitivity of joint moments to changes in $F_{m}^{0}$, $l_{t}^{s}$, $l_{m}^{o}$ and $\alpha_{o}$ parameters of the Hill-type muscle model. Based on (3), the formulation of Hill-type muscle models, $F_{m}^{0}$ linearly affects the joint moments. However, $l_{t}^{s}$, $l_{m}^{o}$ and *α_o_* have a non-linear relationship with muscle force production (refer to Appendix A for Hill model description). Unsurprisingly, moments across many muscle-DoF pairs were most sensitive to $F_{m}^{0}$ with $l_{t}^{s}$ being the next most significant parameters. Our observations presented in Section III-D2 agree with the sensitivity analysis by Charles *et al.* [31]. Charles *et al.* observed that muscles with larger $l_{t}^{s}$ compared to their $l_{m}^{o}$ had their moments more *s* sensitive to $l_{t}^{s}$ than $F_{m}^{0}$. In Table 2 we reported the ratios of $\frac{l_{t}^{s}}{l_{m}^{s}}$. Muscles with smaller ratios (< 1.0) for $\frac{l_{t}^{s}}{l_{m}^{o}}$ have moments more sensitive to $F_{m}^{0}$ and muscles larger $\frac{l_{t}^{s}}{l_{m}^{o}}$ > 1.0 ratios are more sensitive to $l_{t}^{s}$. This in accordance with the previous studies not only in mouse [31] but also for chimpanzees [65] and humans [48], [66].

The same applied to the forelimb muscles as well. Although, in the forelimb we have very few muscles whose ratios of $\frac{l_{t}^{s}}{l_{m}^{o}}$ is greater than 1.0. The validation of our sensitivity analysis for the hindlimb muscle-joint pairs allows us to increase the confidence levels of our observations made for forelimb muscles.

Overall, with our comprehensive analysis we have highlighted the most important parameters in the model. This allows future researchers to identify and work with these important parameters more critically. The identification of the critical parameters also allows for adapting and fine tuning the model in case of numerical optimization for specific behaviors.

### E. MODEL LIMITATIONS

In this work, we present the most complete musculoskeletal model of the mouse. Yet, as with any model, the model construction was possible only because of some simplifications and assumptions. The skeletal model of the mice was developed from anatomical references rather than from an actual microCT scan. This allowed us to generate a more generic representation of the mouse skeleton but meant that not all anatomical features on the bones were captured. In future iterations, a skeletal model based on CT scans will further allow for better model validation. Here, we calculated the inertias of the bones using a bounding box method and assumed a uniform density of water along the body. We did not consider air cavities with different density in the lungs and head in our model. This introduces variation of inertial properties of certain bones and the overall center-of-mass of the mouse musculoskeletal model. Joint rotations were identified manually in this model and use joints such as revolute or spherical joints to represent the different DoFs in the model. However, in animals, joints are more complex and often difficult to model. Any studies that require validated joint movements should try to extend the current version of the model by including the necessary complexities in joint modeling for the particular study.

For the hindlimb muscles, we were able to build on, compare and validate our model with the previous mouse [31] and rat [35] hindlimb models. However, similar validation was not possible for the forelimb. Because of the lack of previous forelimb studies, we had to estimate both the attachment points and the muscle parameters ($F_{m}^{0}$, $l_{m}^{o}$, $l_{t}^{s}$ and $\alpha_{o}$). The comprehensive sensitivity analysis presented in this work provides information about the important parameters to be critically explored in the model. Further experimental measurements from the mouse forelimb are necessary to improve the forelimb model.

### F. MODEL USE AND FUTURE WORK

A musculoskeletal model complements animal experiments by providing the data (EMG, afferent firings, interactions forces between the body and the environment) that is challenging to collect, and they are essential to setup and study closed-loop neuromechanical simulations (Fig. 1). The whole-body model of the mouse presented in this work has the necessary components to contribute to both.

Locomotion is a result of whole-body movement with the neural circuits integrating feed-forward and sensory-driven strategies to generate the necessary muscle activation signals. The limb musculature modeled and analyzed in this work presents an excellent platform to use the model to setup predictive simulations to connect computational models of neural circuits to drive the musculoskeletal model. Alternatively, inverse kinematics and inverse dynamics approaches can be used to estimate kinematics (e.g., joint angles), kinetics (e.g., joint moments) and proprioceptive sensory information (e.g., activities of muscle spindles and Golgi tendon organs) from experimental whole-body trajectories made possible now by markerless pose estimation methods such as DeepLabCut [67].

The majority of the previous locomotion studies in mouse have been limited to straight forward locomotion. Exploring other locomotor regimes such as turning is now experimentally possible [68] and the current 3D model has the necessary DoFs to replicate similar behaviors in simulation. The current model also allows to study motor behaviors that does not involve the whole-body movements like reaching and grasping.

While the possible use cases of our model are plenty, it still is only a preliminary step towards a more robust computational model. As mice are one of the most significant experimental animal models to study normal and pathological motor behaviors, it is extremely important to develop computational models that can complement these studies.

The open-source and modular musculoskeletal model presented here offers an opportunity for a community driven approach that can collectively improve and rigorously validate the different components of the model with experimental data. In future iterations of the model, a systematic identification the full set of forelimb muscles along with the spinal muscles will increase the usability of the model in even more complex scenarios and grow towards a more complete model like the ones available for humans.

## V. CODE AVAILABILITY

All the resources and code for the development and analysis of the mouse model can be found at https://gitlab.com/paper_submissions/mouse_biomechanics_paper

## Figures and Tables

**TABLE 1.** Mouse skeletal segments grouping, joint names and joint ranges.

Group	Bones	Joint	Degree-of-freedom (DoF)	Range-of-motion (degree)	Model	Source
Hindlimb (left/right)	Femur	Hip	extension-flexion	[−30.0, 50.0]	Mouse	[31]
			abduction-adduction	[−40.0, 20.0]	Rat	[35]
			extemal-intemal rotation	[−10.0, 30.0]	Rat	[35]
	Tibia	Knee	flexion-extension	[−145.0, −40.0]	Mouse	[31]
	Tarsus	Ankle	plantarflexion-dorsiflexion	[−50.0, −50.0]	Mouse	[31]
			inversion-eversion	[−30.0, 50.0]	Rat	[35]
			abduction-adduction	[−10.0, 30.0]	Rat	[35]
	MetaTarsus{1–5}	MTPU-5}	extension-flexion	[−5.0, −5.0]	-	
	Phalange{1–5}	Phalange {1–5}	extension-flexion	[−5.0, 5.0]	-	

Forelimb (left/right)	Humerus	Shoulder	retraction-protraction	[−45.0, 45.0]	-	
			abduction-adduction	[−36.0, 3.0]	Rat	[36]
			extemal-intemal rotation	[−40.0, 14.0]	Rat	[36]
	Ulna	Elbow	extension-flexion	[60.0, 153.0]	Rat	[36]
	Radius	Elbow	supination-pronation	[−5.0, 30.0]	Rat	[36]
	Carpus	Wrist	extension-flexion	[−20.0, 50.0]	Rat	[36]
			abduction-adduction	[−10.0, 10.0]		
	MetaCarpus {1–5}	MTC{1–5}	extension-flexion	[−5.0, 5.0]		
	Phalange	Phalange {1–5}	extension-flexion	[−5.0, 5.0]		

Pelvis	Pelvis	Pelvis	-		-	

Spine	Lumbar{1–6}	Lumbar{1–6}	flexion-extension	[−6.0, 6.0]	Mouse	[37]
			lateral bending	[−5.0, 5.0]		
	Thoracic {1–6}	Thoracic{ 1–6}	flexion-extension	[−5.0, 5.0]	Mouse	[37]
			lateral bending	[−5.0, 5.0]		
	Cervicali 1–6}	Cervical{ 1–6}	flexion-extension	[−9.0, 9.0]	Mouse	[38]
			lateral bending	[−5.0, 5.0]		

Head	Upper jaw	Head	flexion-extension	[−50.0, 50.0]	-	
	Lower jaw	Jaw	flexion-extension	[0.0, 30.0]	-	

Tail	Tail{1–23}	Tail{1–23}	flexion-extension	[−10.0, 10.0]	-	
			lateral bending	[−10.0, 10.0]	-	

The skeletal segments are grouped into six sparse groups to fully describe the model. Bones named {1-*n*} indicate *n* bones of the same name suffixed by its appropriate numeric position. A joint is formed between a parent and child (i.e., the bone that undergoes a transformation when the joint is rotated) bone. The table describes only the child bone for each joint. Degree-of-freedom (DoF) indicate the independent DoFs the joint has in the model. Joint range-of-motion for each DoF have been identified from several existing studies (see column ’Model’ and ’Source’). For joints where the information is not available for mice, we have resorted to works that are based on rats instead.

**TABLE 2.** Hindlimb and forelimb muscle parameters.

Muscle-tendon Unit	Abbreviation	$F_{m}^{0}$^(a)^ (N)	$l_{m}^{o}$^(b)^ (mm)	$l_{t}^{s}$^(c)^ (mm)	$\alpha^{o}$^(d)^ (deg)	$\frac{l_{t}^{s}}{l_{m}^{o}}$ ^(e)^	Group^(f)^
HINDLIMB							

Adductor brevis	AB	0.23	9.5	2.66	0.0	0.28	Hip
Adductor longus	AL	0.4	11.9	4.15	0.0	0.35	
Adductor magnus	AM	0.61	12.2	4.85	0.0	0.4	
Biceps femoris anterior	BFA	0.88	17.84	5.97	0.0	0.33	
Caudofemoralis	CF	0.55	17.85	4.82	0.0	0.27	
Gemellus	GEM	0.18	3.33	0.02	0.0	0.01	
Gluteus maximus(dorsal)	GM(dr)	0.94	14.99	5.75	20.42	0.38	
Gluteus maximus(mid)	GM(mi)	1.03	14.28	5.5	20.42	0.38	
Gluteus maximus(ventral)	GM(ve)	1.05	14.59	5.61	20.42	0.38	
Iliacus	ILI	0.55	9.67	3.1	0.0	0.32	
Obturator extemus	OE	0.09	4.37	1.71	0.0	0.39	
Obturator internus	OI	0.31	6.3	0.72	0.0	0.12	
Pectineus	PECT	0.36	4.27	2.79	15.18	0.65	
Psoas major	PMA	1.34	8.01	5.76	15.54	0.72	
Psoas minor	PMI	1.09	6.79	4.58	12.57	0.67	
Quadratus femoris	QF	2.03	4.99	1.18	0.0	0.24	

Biceps femoris posterior(cranial)	BFP(cr)	0.72	16.46	8.02	0.0	0.49	Hip-Knee
Biceps femoris posterior(mid)	BFP(mi)	0.73	16.9	8.08	0.0	0.48	
Biceps femoris posterior(caudal)	BFP(ca)	0.61	19.81	6.72	0.0	0.34	
Gracilis anterior	GA	0.4	14.76	10.16	0.0	0.69	
Gracilis posterior	GP	0.34	15.92	7.68	0.0	0.48	
Rectus femoris	RF	4.16	9.87	15.77	15.89	1.6	
Semimembranosus	SM	1.92	20.14	7.07	0.0	0.35	
Semitendinosus	ST	1.3	19.1	8.25	0.0	0.43	

Popliteus	POP	0.31	3.3	3.26	0.0	0.99	Knee
Vastus intermedius	VI	0.37	10.08	11.68	10.92	1.16	
Vastus lateralis	VL	2.83	11.18	12.07	15.53	1.08	
Vastus medialis	VM	1.1	10.77	12.67	16.15	1.18	

Lateral gastrocnemius	LG	3.78	6.97	17.89	17.28	2.57	Ankle-Knee
Medial gastrocnemius	MG	1.75	7.29	18.5	14.24	2.54	
Plantaris	PLANT	0.88	5.75	20.23	17.1	3.52	

Extensor digitorum longus	EDL	0.37	8.12	30.42	12.39	3.74	Ankle
Extensor hallucis longus	EHL	0.07	7.71	23.32	9.56	3.02	
Flexor digitorum longus	FDL	1.9	5.71	36.6	15.2	6.41	
Peroneus brevis	PB	0.4	3.04	13.33	11.46	4.39	
Peroneus digit! quarti	PDQA	0.11	5.05	30.31	12.42	6.0	
Peroneus digiti quinti	PDQI	0.1	4.72	25.53	9.44	5.41	
Peroneus longus	PL	0.65	5.21	19.4	14.9	3.72	
Peroneus tertius	PT	0.46	4.78	15.81	12.46	3.31	
Soleus	SOL	0.59	4.5	10.53	11.43	2.34	
Tibialis anterior	TA	2.42	6.61	15.92	16.58	2.41	
Tibialis posterior	TP	0.55	4.72	19.71	15.44	4.18	

FORELIMB							

Coracobrachialis	COR	0.28	5.18	4.64	13.85	0.9	Shoulder

Biceps brachii long head	BBL	0.78	11.63	7.52	21.68	0.65	Elbow-Shoulder
Triceps brachii lateral head	TBL	0.71	8.17	4.53	23.41	0.55	

Anconeus	AN	0.15	1.42	1.49	24.65	1.05	Elbow
Biceps brachii short head	BBS	0.84	9.08	6.07	28.27	0.67	
Brachialis	BRA	0.25	10.06	5.58	9.89	0.56	
Pronator teres	PTE	0.55	2.75	4.96	26.17	1.81	
Triceps brachii medial head	TBM	0.45	4.93	6.43	30.33	1.3	
Triceps brachii long head*	TBO	4.65	7.01	4.88	41.88	0.7	

Extensor carpi radialis brevis	ECRB	0.35	14.45	7.71	29.49	0.53	Elbow-Wrist
Extensor carpi radialis longus	ECRL	0.38	13.86	7.29	26.4	0.53	
Extensor carpi ulnaris	ECU	0.52	7.67	10.84	20.8	1.41	
Extensor indicis proprius 1	EIP1	0.07	5.05	2.99	19.0	0.59	
Extensor indicis proprius 2	EIP2	0.07	5.27	3.3	19.0	0.63	
Flexor carpi radialis	FCR	0.38	8.92	10.74	15.02	1.2	
Flexor carpi ulnaris	FCU	0.69	8.28	10.29	20.95	1.24	
Palmaris longus	PLO	0.27	10.9	9.12	24.65	0.84	

(a) Maximum isometric force of the muscle. Hindlimb muscle forces are the same as reported in [31]. Forelimb muscle forces are computed by multiplying the physiological cross-sectional area (PCSA) reported in [54] by isometric stress (σ = 0.3 N/mm^2^) [31], [60].

(b) Optimal fiber length for hindlimb scaled to the current model from [31] using the scaling based on [53] and forelimb scaled to the current model [54].

(c) Tendon slack length is obtained in the same way as described for the optimal fiber length.

(d) Pennation angle for hindlimb is obtained from [31] and for forelimb from [54].

(e) Ratio of optimal fiber length and tendon slack length.

(f) Sorting of muscles into groups based on the joints they span. For example, Biceps femoris posterior (BFP) belongs to Hip-Knee group as it spans over one or more hip and knee joints. The same order is used in the rest of this work.

## APPENDIX A HILL-TYPE MUSCLE MODEL

The force produced by the contractile and parallel elements was modeled as,

(3)
$$F=F_{m}^{0}\left(a(t)f_{l}\left({\tilde{l}}_{m}\right)f_{v}\left({\tilde{v}}_{m}\right)+f_{PE}\left({\tilde{l}}_{m}\right)+\beta {\tilde{v}}_{m}\right)$$

$F_{m}^{0}$ is the maximum isometric force the muscle is capable of producing. *a*(*t*) is the muscle activation. $f_{l}\left({\tilde{l}}_{m}\right)$ defines the force-length relationship curve during isometric contractions with ${\tilde{l}}_{m}$ being the normalized muscle-fiber length. $f_{v}\left({\tilde{v}}_{m}\right)$ defines the force-velocity relationship curve during isotonic contractions with ${\tilde{v}}_{m}$ being the normalized muscle-fiber length. Passive forces are generated in the muscle when stretched beyond a threshold length, this is represented by the passive-force-length given by $f_{PE}\left({\tilde{l}}_{m}\right).\;\beta {\tilde{v}}_{m}$ represents the additional damping in the muscle with *β* being the damping coefficient, set to 0.1 by default.

The activation dynamics (*a*(*t*)) and passive-force-length (*f_PE_*) are described based on the implementation from Opensim [69].

(4)
$$\frac{da(t)}{dt}=\frac{u(t)-a(t)}{\tau_{act}}$$

where *u*(*t*) is the neural-excitation input signal to the muscles bounded within a range of 0 and 1, and *τ_act_* is the timeconstant (Note that we used a constant value for $\tau_{act}$ instead of varying it as a function of *u*(*t*) and *a*(*t*) as described by Thelen [69].)

(5)
$$f_{PE}\left({\tilde{l}}_{m}\right)=\frac{\exp\left(k_{PE}\left({\tilde{l}}_{m}-1\right)/\epsilon_{0}^{m}\right)-1}{\exp\left(k_{PE}\right)-1}$$

The active-force-length ($f_{l}$) and force-velocity ($f_{v}$) relationships described below are based on the description by De Groote *et al.* in [70]

(6)
$$f_{l}\left({\tilde{l}}_{m}\right)=\sum_{i=1}^{3}b_{1i}\exp{\left[\frac{-0.5\left({\tilde{l}}_{m}-b_{2i}\right)}{b_{3i}+b_{4i}{\tilde{l}}_{m}}\right]}^{2}$$

(7)
$$f_{v}\left({\tilde{v}}_{m}\right)=d_{1}\log\left[\left(d_{2}{\tilde{v}}_{m}+d_{3}\right)+\sqrt{\left({\left(d_{2}{\tilde{v}}_{m}+d_{3}\right)}^{2}+1\right))}\right]\;\;+d_{4}$$

Since, the tendon is assumed to be rigid and inextensible, the force along the tendon (*F_t_*) equates to the force produced by the contractile element described in (3) as,

(8)
$$F_{t}=F\cos(\alpha )$$

## APPENDIX B HINDLIMB MUSCLE PARAMETER SCALING

Optimal muscle-fiber length ($l_{m}^{o}$)and muscle-tendon slack length ($l_{t}^{s}$) were scaled from Charles model [31] (referred to as reference model) to our model (referred to as target model). To do the scaling, we used the algorithm proposed by Modenese *et al.* in [53], steps for which are described below,

- In the reference model, identify the *N_q_* degrees-of-freedom spanned by the muscle. Discretize each joint into $n_{dof}$ poses to create a set of $n={\left(n_{dof}\right)}^{N_{q}}$ possible joint pose combinations.
- In the reference model, for each pose 1,2,3,…,n, compute a vector of pennation angles ($\alpha_{\text{ref}}$), normalized muscle-fiber length (${\tilde{\text{l}}}_{\text{m},\text{ref}}$) and normalized muscle-tendon lengths (${\tilde{l}}_{t,\mathrm{ref}}$)
- In the target model, for the same *n* poses, compute a vector of muscle-tendon lengths ($l_{\text{mt},\text{targ}}$)
- Solve for muscle-tendon slack length $l_{t}^{s}$ in the target model by solving (9) using a least-squares-like numerical method.

(9)
$${\left[\begin{array}{c} l_{mt}^{1}\\ l_{mt}^{2}\\ \cdot \\ \cdot \\ l_{mt}^{n} \end{array}\right]}_{targ}={\left[\begin{array}{cc} {\tilde{l}}_{m}^{1}\cos(\alpha ) & {\tilde{l}}_{t}^{1}\\ {\tilde{l}}_{m}^{2}\cos(\alpha ) & {\tilde{l}}_{t}^{2}\\ \cdot & \cdot \\ \cdot & \cdot \\ {\tilde{l}}_{m}^{n}\cos(\alpha ) & {\tilde{l}}_{t}^{n} \end{array}\right]}_{ref}{\left[\begin{array}{c} l_{m}^{o}\\ l_{t}^{s} \end{array}\right]}_{targ}$$

We solved (9) using the least squares method implemented in Scipy *v1.70*.

## APPENDIX C MUSCLE-TENDON SLACK LENGTH ESTIMATION

Muscle-tendon slack length for the forelimb muscles were estimated based on the modified algorithm proposed by Manal and Buchanan in [55].

For the purpose of estimating the muscle-tendon slack length, we consider the tendons to be elastic and non-rigid whose force-tendon length $\left(f_{t}\left({\tilde{l}}_{t}\right)\right)$ (with ${\tilde{l}}_{t}=\frac{l_{t}}{l_{t}^{s}}$ being the normalized muscle-tendon length) characteristics are defined by the formulation by De Groote *et al.* in [70] as,

(10)
$$f_{t}\left({\tilde{l}}_{t}\right)=c_{1}\exp\left[k_{T}\left({\tilde{l}}_{t}-c_{2}\right)\right]-c_{3}$$

**TABLE 3.** Hill-type muscle model parameters.

Activation dynamics	$\tau_{\text{act}}$(s)	0.001
Active force-length	b_11_	0.815
	b_21_	1.055
	b_31_	0.162
	b_41_	0.063
	b_12_	0.433
	b_22_	0.717
	b_32_	−0.030
	b_42_	0.200
	b_13_	0.100
	b_23_	1.00
	b_33_	0.354
	b_34_	0.0

Active force-velocity	d_1_	−0.318
	d_2_	−8.149
	d_3_	−0.374
	d_4_	0.886

Passive force-length	k_PE_	4.0
	ϵ_0_	0.600

Tendon force-length	k_T_	35
	c_1_	0.200
	c_2_	0.995
	c_3_	0.250
Damping	*β*	0.100

Substituting (10) in (8),

(11)
$$F_{m}^{0}f_{t}\left({\tilde{l}}_{t}\right)=F_{m}^{0}\left(a(t)f_{l}\left({\tilde{l}}_{m}\right)f_{v}\left({\tilde{v}}_{m}\right)+f_{PE}\left({\tilde{l}}_{m}\right)+\beta {\tilde{v}}_{m}\right)$$

The total length of the muscle-tendon unit is,

(12)
$$l_{mt}=l_{m}\cos(\alpha )+l_{t}$$

Equation 12 can be expressed in terms of normalized muscle-fiber length (${\tilde{l}}_{m}$) and normalized tendon (${\tilde{l}}_{t}$) as,

(13)
$$l_{mt}={\tilde{l}}_{m}l_{m}^{o}\cos(\alpha )+{\tilde{l}}_{t}l_{t}^{s}$$

From (13) $l_{t}^{s}$ can be written as,

(14)
$$l_{t}^{s}=\frac{l_{mt}-{\tilde{l}}_{m}l_{m}^{o}\cos(\alpha )}{{\tilde{l}}_{t}}$$

Considering a maximal activation (*a*(*t*) = 1.0) under isometric conditions (${\tilde{v}}_{m}$ = 0.0), from (10), (11) and (14), $l_{t}^{s}$ can be written as,

(15)
$$c_{1}\exp\left[k_{T}\left({\tilde{l}}_{t}-c_{2}\right)\right]-c_{3}\;\;=\left(f_{l}\left({\tilde{l}}_{m}\right)+f_{PE}\left({\tilde{l}}_{m}\right)\right)\cos(\alpha )$$

(16)
$${\tilde{l}}_{t}=\frac{c_{2}+\left(\log\left[\left(\left(f_{l}\left({\tilde{l}}_{m}\right)+f_{PE}\left({\tilde{l}}_{m}\right)\right)\cos(\alpha )+c_{3}\right)/c_{1}\right]\right)}{k_{T}}$$

(17)
$$l_{t}^{s}=\frac{l_{mt}-{\tilde{l}}_{m}l_{m}^{o}\cos(\alpha )}{c_{2}+\left(\log\left[\left(\left(f_{l}\left({\tilde{l}}_{m}\right)+f_{PE}\left({\tilde{l}}_{m}\right)\right)\cos(\alpha )+c_{3}\right)/c_{1}\right]\right)/k_{T}}$$

Here we uses the tendon force-length relationship formulated by De Groote *et al.* in [70] instead of the one described Manal and Buchanan in [55].

Given ${\tilde{l}}_{m}$ at $l_{mt}$ and knowing $l_{m}^{o}$, we can compute tendon slack length $l_{t}^{s}$ from (17). But, we do not know ${\tilde{l}}_{m}$ in practice. Manal and Buchanan proposed to use numerical methods to estimate ${\tilde{l}}_{m}$ at short, long and mid range of $l_{mt}$ such that $l_{t}^{s}$ is to be a constant, which was formulated as a minimization problem.

$$\text{minimize}\left[{\left(l_{t}^{s1}-l_{t}^{s2}\right)}^{2}+{\left(l_{t}^{s1}-l_{t}^{s3}\right)}^{2}+{\left(l_{t}^{s2}-l_{t}^{s3}\right)}^{2}\right]\;\text{subject to,}\;{\tilde{l}}_{m}\leq 1.0$$

We extended this formulation by Manal *et al.* for $l_{t}^{s}$ in [55] by including the passive forces ($f_{PE}\left({\tilde{l}}_{m}\right)$) in (17) so that we include the complete force-length curve for estimation. The formalization described above is limited to the muscles’ relationship with only a single degree-of-freedom. We extend this to all the degrees-of-freedom (*ndof*) where the muscle has a significant moment-arm. This is done by estimating ${\tilde{l}}_{m}$ at short, long and mid range of $l_{mt}$ for each degree-of-freedom the muscle influences. The new minimization problem is defined as,

(18)
$$\text{minimize}\;\sum_{n=1}^{ndof}{\left[{\left(l_{t}^{s1}-l_{t}^{s2}\right)}^{2}+{\left(l_{t}^{s1}-l_{t}^{s3}\right)}^{n2}+{\left(l_{t}^{s2}-l_{t}^{s3}\right)}^{2}\right]}_{n}$$

(19)
$$\text{subject to,}\;0.6\leq {\tilde{l}}_{m}\leq 1.4$$

We used differential evolution, a stochastic population based optimization technique implemented in Scipy *v*1.70 to minimize the objective in (18) for each muscle in the forelimb. A population size of 20, maximum iterations of 1000 and relative and absolute tolerances were set to 1e^−10^.
